# Solvent-free and room temperature synthesis of 3-arylquinolines from different anilines and styrene oxide in the presence of Al_2_O_3_/MeSO_3_H

**DOI:** 10.3762/bjoc.13.193

**Published:** 2017-09-20

**Authors:** Hashem Sharghi, Mahdi Aberi, Mohsen Khataminejad, Pezhman Shiri

**Affiliations:** 1 Department of Chemistry, Shiraz University, Shiraz, 71454, I. R. Iran, Tel.: +98 711 2284822; Fax: +98 711 2280926

**Keywords:** 3-arylquinolines, Al_2_O_3_, MeSO_3_H, one-pot reaction, solvent-free conditions

## Abstract

A highly efficient, simple and environmentally friendly synthesis of 3-arylquinolines has been developed in the presence of Al_2_O_3_/MeSO_3_H via one-pot reaction of anilines and styrene oxide. This methodology provides very rapid access to 3-arylquinolines in good to excellent yields under solvent-free conditions at room temperature in air.

## Introduction

Quinoline derivatives have received considerable interest because they are found in numerous natural products with many biological activities. They have also played an important role in medicinal chemistry due to their pharmacological properties [1–4]. Transition metal-catalyzed processes [5–8] and metal-free paths [9–11] are two general approaches for the synthesis of this type of compounds. However, the existing methods suffer from complicated multistep processes, limited availability of substrates, toxic organic solvents, long reaction times, expensive catalyst and low regioselectivity in some cases.

One current area of modern synthetic organic chemistry is the development of powerful and effective practical procedures that minimize the requisite time, temperature, labour, and cost for the desired transformations [12–13]. The tandem reaction of anilines with styrene oxide via C–C cleavage is the efficient synthetic route to quinolones [1]. The reaction was performed using FeCl_3_ as catalyst in 1,4-dioxane as solvent at 110 °C for 12 h. According to the significance of this progress, we have decided to re-optimize it. The mixture of Al_2_O_3_ and MeSO_3_H has been previously used as an effective and mild reagent for organic transformations [14].

**Scheme 1:**
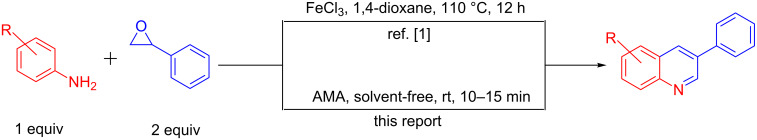
Synthesis of 3-arylquinolines from anilines and styrene oxide. AMA = Al_2_O_3_/methanesulfonic acid

In continuation of our studies to develop new synthetic methods for heterocycles [15–19], herein, we disclose a novel route to the synthesis of 3-arylquinolines from aniline derivatives and styrene oxide at room temperature under solvent-free conditions (Scheme 1).

Scheme 1 briefly compares the procedure reported by Wang and co-workers and our method. As it can be seen, the reaction can be performed under very short reaction time and low temperature.

## Results and Discussion

To exploit optimized conditions for the synthesis of quinolines, the reaction of 3,4-dimethylaniline (**1**, 1.0 mmol) and styrene oxide (**2**, 2.0 mmol) in an open atmosphere was chosen as a model reaction (Table 1).

Control experiments showed that in the absence of Al_2_O_3_ and MeSO_3_H, no quinoline **3a** was observed (Table 1, entry 1). The results also showed the importance of using both of Al_2_O_3_ and MeSO_3_H. In the absence of MeSO_3_H no product was obtained (Table 1, entry 2). In the presence of MeSO_3_H, 6,7-dimethyl-3-phenylquinoline (**3a**) was obtained in 75% yield (Table 1, entry 3). Finally, the best result was obtained using a mixture of Al_2_O_3_ (0.1 g) and MeSO_3_H (0.3 mL) under solvent-free conditions at room temperature for 10 min (Table 1, entry 6). Various anilines with electron-withdrawing and electron-donating functional groups such as *o*-Me, *m*-Me, *p*-Me, *m*-Et, *p*-MeO, *m*-Br, *o*-Cl, 3,4-dimethyl and *p*-OEt were treated with styrene oxide to form the desired products (Table 2). In continuation of our study, aliphatic epoxides were also checked; unfortunately, they were not applicable for the preparation of quinolines. All novel and known compounds were characterized by their melting points, IR, ^1^H NMR, ^13^C NMR and mass spectra.

**Table 1:** Optimization studies for the synthesis of 6,7-dimethyl-3-phenylquinoline (**3a**) from 3,4-dimethylaniline (**1a**, 1.0 mmol) with styrene oxide (**2**, 2.0 mmol) in open air.

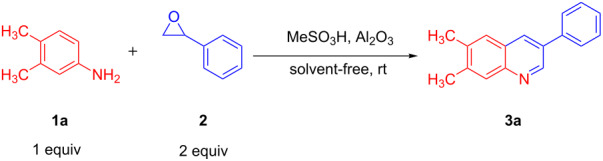				

Entry	Catalyst	Conditions	Time (min)	Yield (%)^a^

1	None	solvent-free/rt	60	no reaction
2	Al_2_O_3_ (0.1 g)	solvent-free/rt	60	no reaction
3	MeSO_3_H (0.3 mL)	solvent-free/rt	10	75
4	Al_2_O_3_ (0.1 g) + MeSO_3_H (0.1 mL)	solvent-free/rt	10	35
5	Al_2_O_3_ (0.1 g) + MeSO_3_H (0.2 mL)	solvent-free/rt	10	79
6	Al_2_O_3_ (0.1 g) + MeSO_3_H (0.3 mL)	solvent-free/rt	10	91
7	Al_2_O_3_ (0.2 g) + MeSO_3_H (0.3 mL)	solvent-free/rt	10	85

^a^Isolated yield.

**Table 2:** One-pot synthesis of 3-arylquinolines from the reaction of different anilines (1.0 mmol) with styrene oxide (2.0 mmol) in the presence of Al_2_O_3_ and MeSO_3_H at room temperature under solvent-free conditions.

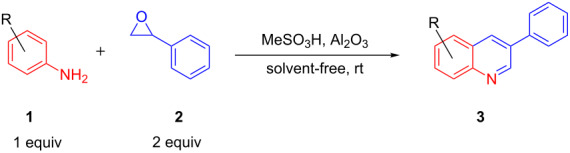				

Entry	Aniline	Product	Time (min)	Yield (%)^a^

1	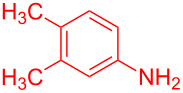 **1a**	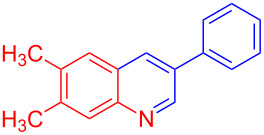 **3a**	10	91
2	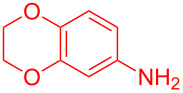 **1b**	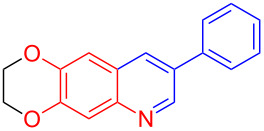 **3b**	12	89
3	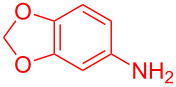 **1c**	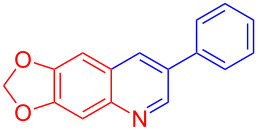 **3c**	12	88
4	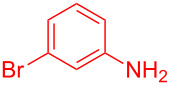 **1d**	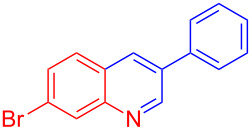 **3d**	15	86
5	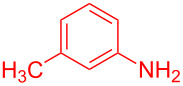 **1e**	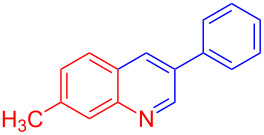 **3e**	10	85
6	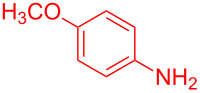 **1f**	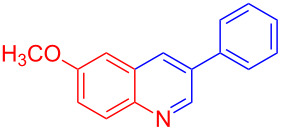 **3f**	15	84
7	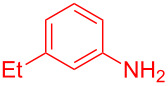 **1g**	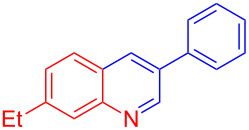 **3g**	10	83
8	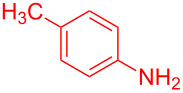 **1h**	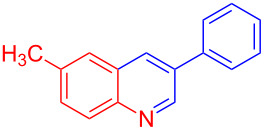 **3h**	10	82
9	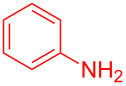 **1i**	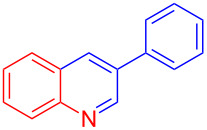 **3i**	12	81
10	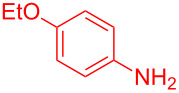 **1j**	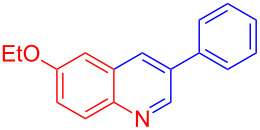 **3j**	15	84
11	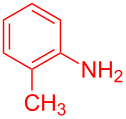 **1k**	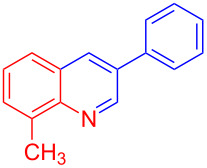 **3k**	10	82
12	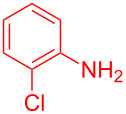 **1l**	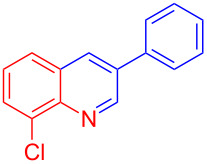 **3l**	15	80

^a^Isolated yield.

**Figure 1:**
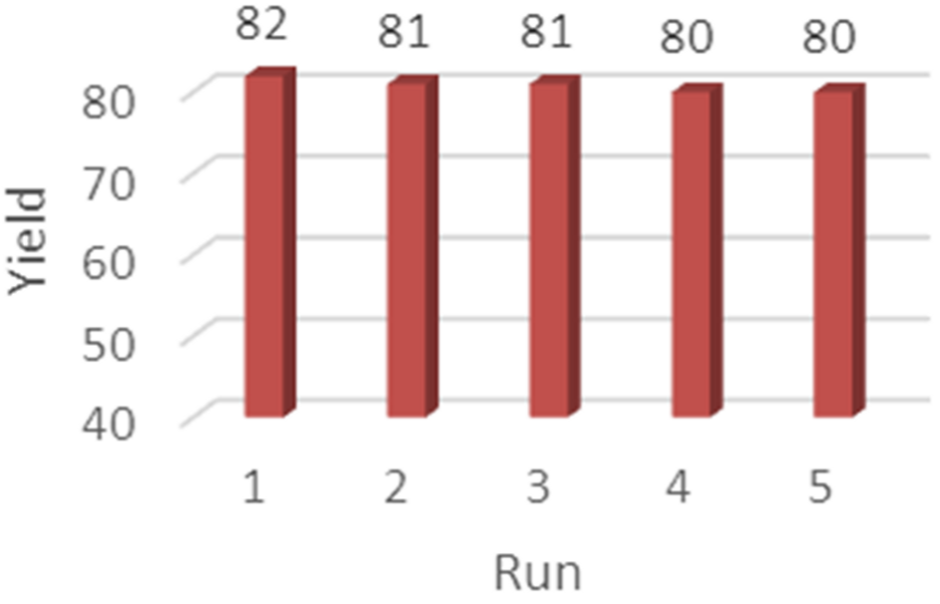
Investigation of the reusability of Al_2_O_3_.

In order to recover Al_2_O_3_, the mixture was diluted with ethyl acetate and filtered. The solid on the filter paper was washed by ethyl acetate and evaporated. It should be noted that only Al_2_O_3_ was reused and it is necessary to add MeSO_3_H again for each cycle with recovered Al_2_O_3_. The recycled catalyst could be reused five times without any significant loss in activity (Figure 1).

## Conclusion

In conclusion, Al_2_O_3_ and MeSO_3_H exhibited an excellent reactivity in the one-pot synthesis of 3-arylquinolines using anilines and styrene oxide. The methodology has the advantages of good to excellent yields, readily available starting materials, short reaction time, mild and solvent-free conditions. The method utilizes nonexpensive reagents and starting materials, as well. Further work is in progress to extend the scope and to investigate mechanism aspects of this reaction.

## Experimental

### Instrumentation, analysis and starting material

Starting materials and solvents were purchased from Aldrich, Fluka, and Merck. IR spectra were obtained using a Shimadzu Fourier transform infrared (FTIR) 8300 spectrophotometer. Melting points were determined in open capillary tubes in a Büchi-535 circulating oil melting point apparatus. Mass spectra were determined on a Shimadzu GCMS-QP 1000 EX instrument at 70 or 20 eV. NMR spectra were recorded on a Bruker Avance DPX-250 (^1^H NMR 250 MHz and ^13^C NMR 62.9 MHz) spectrometer in pure deuterated solvents with tetramethylsilane (TMS) as an internal standard. The used methanesulfonic acid 98% and acidic alumina (Al_2_O_3_) type 540 C were purchased from Fluka. The elemental analyses were performed with a Thermo Finnigan CHNS-O analyzer, 1112 series. The purity determination of the substrates and reaction monitoring were accomplished by TLC on silica gel PolyGram SILG/UV 254 plates. Column chromatography was carried out on short columns of silica gel 60 (70–230 mesh) in glass columns.

### General procedure for the synthesis of quinolines in the presence of Al_2_O_3_/MeSO_3_H

Aniline (1.0 mmol) and styrene oxide (2.0 mmol) were added to a mixture of MeSO_3_H (0.3 mL) and Al_2_O_3_ (0.1 g). The mixture was stirred at room temperature in solvent-free conditions for the period of time reported in Table 2. After completion of the reaction, the mixture was diluted with ethyl acetate, and filtered. The filtrate was washed with a solution of NaHCO_3_ (5%; 3 × 30 mL) and then 30 mL deionized water. The solution was dried over magnesium sulfate; the solvent was evaporated to give the crude product, which was purified by silica gel column chromatography employing *n*-hexane/ethyl acetate (10:1) as eluent.

## Supporting Information

File 1 Additional experimental and analytical data and NMR spectra.
