# Supplementary material for: Solvent-free and room temperature synthesis of 3-arylquinolines from different anilines and styrene oxide in the presence of Al2O3/MeSO3H
Source: Beilstein J Org Chem. 2017 Sep 20;13:1977–81. doi: 10.3762/bjoc.13.193 (PMC5629378; doi:10.3762/bjoc.13.193)
Supplement: File 1 — Additional experimental and analytical data and NMR spectra. [file Beilstein_J_Org_Chem-13-1977-s001.pdf]

Supporting Information

for

Solvent-free and room temperature synthesis of 3-arylquinolines from different anilines and styrene oxide in the presence of Al_2O_3/$MeSO_3H$

Hashem Sharghi*, Mahdi Aberi, Mohsen Khataminejad, and Pezhman Shiri

Address: Department of Chemistry, Shiraz University, Shiraz, 71454, I. R. Iran, Tel.: +98 711 2284822; Fax: +98 711 2280926

Email: Hashem Sharghi*- shashem@susc.ac.ir

*Corresponding author

Additional experimental and analytical data and NMR spectra

Experimental

Instrumentation, analysis and starting material:

Starting materials and solvents were purchased from Aldrich, Fluka, and Merck. IR spectra were obtained using a Shimadzu Fourier transform infrared (FTIR) 8300 spectrophotometer. Melting points determined in open capillary tubes in a Büchi-535 circulating oil melting point apparatus. Mass spectra were determined on a Shimadzu GCMS-QP 1000 EX instrument at 70 or 20 ev. NMR spectra were recorded on a Bruker Avance DPX-250 (^{1}H NMR 250 MHz and ^{13}C NMR 62.9 MHz) spectrometer in pure deuterated solvents with tetramethylsilane (TMS) as an internal standard. The used methanesulfonic acid 98% and acidic alumina (Al_2O_3) type 540 C were purchased from Fluka. Elemental analyses were performed with a Thermo Finnigan CHNS-O analyzer, 1112 series. The purity determination of the substrates and reaction monitoring were accomplished by TLC on silica gel PolyGram SILG/UV 254 plates. Column chromatography was carried out on short columns of silica gel 60 (70–230 mesh) in glass columns.

General procedure for the synthesis of quinolines in the presence of Al_2O_3/$MeSO_3H$ (AMA)

Aniline (1.0 mmol) and styrene oxide (2.0 mmol) was added to a mixture of $MeSO_3H$ (0.3 mL) and Al_2O_3 (0.1 g). The mixture was stirred at room temperature in solvent free conditions for the period of time reported in Table 2. After completion of the reaction, the mixture was diluted with ethyl acetate, and filtered. The filtrate was washed with a solution of $NaHCO_3$ (5%; 3 × 30 mL) and then 30 mL deionized water. The solution was dried over magnesium

sulfate; the solvent was evaporated to give the crude product, which was purified by silica gel column chromatography employing *n*-hexane/ethyl acetate (10:1) as eluent.

Physical data of the compounds isolated

6,7-Dimethyl-3-phenylquinoline (3a)

Pale yellow solid; mp 122–123 °C. IR (KBr): 694, 763, 864, 910, 1026, 1226, 1342, 1450, 1488, 1550, 2939, 2977, 3024 cm^{-1}. ^{1}H NMR ($CDCl_3$, 250 MHz): δ 2.47 (s, 3 H), 2.50 (s, 3 H), 7.42-7.55 (m, 3 H), 7.62 (s, 1 H), 7.68-7.73 (m, 2 H), 7.90 (s, 1 H),8.20 (d, *J* = 2.0 Hz, 1 H),9.08 (d, *J* = 2.2 Hz, 1 H). ^{13}C NMR ($CDCl_3$, 62.9 MHz): δ 20.1, 20.5, 126.6, 127.2, 127.3, 127.8, 128.4, 129.1, 132.3, 133.0, 137.0, 139.8, 148.8. Mass m/z (%): 235 (M^++2, 12.5), 234 (M^++1, 60.6), 233 (M^+, 100.0), 218 (33.1), 105 (28.0), 83 (33.4), 57 (70.3). Anal. Calcd for $C_{17}H_{15}N$ (233.312): C, 87.52; H, 6.48; N, 6.00; found: C, 87.46; H, 6.43; N, 6.08.

8-Phenyl-2,3-dihydro-[1,4]dioxino[2,3-*g*]quinoline (3b)

Gray solid; mp 151–152 °C. IR (KBr): 694, 740, 910, 1026, 1149, 1234, 1280, 1350, 1450, 1496, 2869, 2923, 3008 cm^{-1}. ^{1}H NMR ($CDCl_3$, 250 MHz): δ 4.37 (s, 4 H), 7.24 (s, 1 H), 7.36-7.53 (m, 3 H), 7.56 (s, 1 H), 7.65-7.69 (m, 2 H), 8.09 (d, *J* = 1.0 Hz, 1 H), 8.98 (d, *J* = 1.5 Hz, 1 H). ^{13}C NMR ($CDCl_3$, 62.9 MHz): δ 64.3, 64.4, 112.0, 113.9, 124.0, 127.2, 127.8, 129.1, 131.6, 132.1, 138.1, 143.8, 144.7, 146.8, 148.2. Mass m/z (%):265 (M^++2, 3.4), 264 (M^++1, 13.6), 263 (M^+, 21.3), 179 (8.6), 149 (10.8), 129 (10.7), 97 (19.1), 69 (100.0). Anal. Calcd for $C_{17}H_{13}NO_2$ (263.294): C, 77.55; H, 4.98; N, 5.32; found: C, 77.49; H, 5.06; N, 5.39.

7-Phenyl-[1,3]dioxolo[4,5-g]quinoline [1] (3c)

Pale brown solid; mp 125–127 °C. IR (KBr): 694, 756, 848, 925, 1033, 1080, 1149, 1234, 1334, 1465, 1612, 2900, 3047 cm^{-1}. ^1H NMR ($CDCl_3$, 250 MHz): δ 6.10 (s, 2 H), 7.08 (s, 1 H), 7.40-7.53 (m, 4 H), 7.64-7.68 (m, 2 H), 8.09 (d, *J* = 2.2 Hz, 1 H), 8.95 (d, *J* = 2.2 Hz, 1 H). ^{13}C NMR ($CDCl_3$, 62.9 MHz): δ 101.8, 102.8, 105.5, 125.03, 127.1, 127.8, 129.1, 132.2, 137.9, 145.5, 147.3, 148.1, 150.6. Mass m/z (%): 251 (M^++2, 2.3), 250 (M^++1, 11.0), 249 (M^+, 22.0), 219 (100.0), 190 (14.4), 165 (9.4), 149 (10.5), 115 (8.3), 73 (12.8), 57 (30.7). Anal. Calcd for $C_{16}H_{11}NO_2$ (249.268): C, 77.10; H, 4.45; N, 5.62; found: C, 77.18; H, 4.52; N, 5.55.

7-Bromo-3-phenylquinoline (3d)

Yellow oil. IR (KBr): 694, 756, 802, 910, 948, 1056, 1157, 1334, 1442, 1481, 1589, 2854, 2923, 3039 cm^{-1}. ^1H NMR ($CDCl_3$, 250 MHz): δ 7.39-7.47 (m, 3 H), 7.59-7.72 (m, 4 H), 8.25 (s, 1 H), 8.32 (s, 1 H), 9.10 (s, 1 H). ^{13}C NMR ($CDCl_3$, 62.9 MHz): δ 124.1, 126.7, 127.3, 128.6, 129.3, 129.4, 130.7, 131.1, 134.1, 134.3, 136.9, 146.5, 149.8. Mass m/z (%): 286 (M^++2, 34.6), 285 (M^++1, 100.0), 284 (M^+, 45.3), 203 (28.7), 176 (16.1), 104 (23.7), 57 (16.8).

7-Methyl-3-phenylquinoline [2-5] (3e)

Yellow oil. IR (KBr): 694, 756, 810, 902, 1033, 1141, 1334, 1450, 1496, 1566, 1612, ,2885, 2916, 3024 cm^{-1}. ^1H NMR ($CDCl_3$, 250 MHz): δ 2.87 (s, 3 H), 7.44-7.57 (m, 5 H), 7.72-7.75 (m, 3 H), 8.30 (d, *J* = 2.2 Hz, 1 H), 9.23 (d, *J* = 2.5 Hz, 1 H). ^{13}C NMR ($CDCl_3$, 62.9 MHz): δ 18.2, 126.1, 126.8, 127.4, 128.0, 128.4, 129.2, 129.7, 133.5, 133.6, 136.9, 138.0, 146.4, 148.7. Mass m/z (%):221 (M^++2, 13.9), 220 (M^++1, 77.8), 219 (M^+, 100.0), 189 (10.4), 165 (7.0), 142 (4.0), 115 (6.4), 89 (4.0).

6-Methoxy-3-phenylquinoline [2-5] (3f)

Yellow oil. IR (KBr): 694, 756, 833, 902, 948, 1026, 1118, 1164, 1211, 1242, 1342, 1373, 1458, 1496, 1620, 2931, 2962, 3042 cm^{-1}. ^{1}H NMR ($CDCl_3$, 250 MHz): δ 3.86 (s, 3 H), 7.04 (d, *J* = 2.7 Hz, 1 H), 7.26-7.46 (m, 4 H), 7.59-7.63 (m, 2 H), 7.95 (d, *J* = 9.2 Hz, 1 H), 8.11 (d, *J* = 2.2 Hz, 1 H), 8.94 (d, *J* = 2.2 Hz, 1 H). ^{13}C NMR ($CDCl_3$, 62.9 MHz): δ 55.5, 105.3, 122.3, 127.4, 128.1, 129.1, 130.5, 132.2, 134.1, 138.0, 143.3, 147.3, 158.1. Mass m/z (%): 237 (M^++2, 13.7), 236 (M^++1, 67.0), 235 (M^+, 100.0), 192 (51.7), 165 (16.4), 97 (11.0), 73 (16.5), 57 (44.9).

7-Ethyl-3-phenylquinoline (3g)

Yellow oil. IR (KBr): 694, 756, 817, 902, 1365, 1450, 1566, 1627, 2864, 2931, 2962, 3024 cm^{-1}. ^{1}H NMR ($CDCl_3$, 250 MHz): δ 1.38 (t, *J* = 7.5 Hz, 3 H), 2.90 (q, *J* = 7.5 Hz, 2 H), 7.40-7.55 (m, 4 H), 7.68-7.73 (m, 2 H), 7.81 (d, *J* = 8.5 Hz, 1 H), 7.95 (s, 1 H), 8.28 (d, *J* = 2.2 Hz, 1 H), 9.16 (d, *J* = 2.2 Hz, 1 H). ^{13}C NMR ($CDCl_3$, 62.9 MHz): δ 15.2, 29.1, 126.3, 126.7, 127.3, 127.4, 127.6, 127.8, 128.0, 128.1, 128.4, 129.1, 133.1, 137.9, 146.1, 147.5, 149.6. Mass m/z (%): 235 (M^++2, 10.1), 234 (M^++1, 67.6), 233 (M^+, 95.2), 218 (100.0), 115 (10.1), 77 (11.3).

6-Methyl-3-phenylquinoline [2-5] (3h)

Yellow oil. IR (KBr): 694, 765, 902, 1033, 1375, 1450, 1498, 2916, 3027 cm^{-1}. ^{1}H NMR ($CDCl_3$, 250 MHz): δ 2.49 (s, 3 H), 7.40-7.52 (m, 5 H), 7.64-7.67 (m, 2 H), 8.08-8.11 (m, 2 H), 9.14 (d, *J* = 2.2 Hz, 1 H). ^{13}C NMR ($CDCl_3$, 62.9 MHz): δ 21.7, 126.9, 127.4, 128.1, 128.5, 128.6, 128.8, 129.2, 131.7, 132.5, 133.7, 136.8, 137.9, 146.0, 148.9. Mass m/z (%): 221 (M^++2, 15.4), 220

(M^++1, 90.6), 219 (M^+, 100.0), 189 (10.5), 165 (6.7), 142 (4.0), 115 (7.6), 89 (4.2), 57 (3.0).

3-Phenylquinoline [2-5] (3i)

Yellow oil. IR (KBr): 692, 763, 902, 1026, 1365, 1450, 1496, 1565, 2900, 3001, 3047 cm^{-1}. ^{1}H NMR ($CDCl_3$, 250 MHz): δ 7.44-7.59 (m, 4 H), 7.71-7.74 (m, 3 H), 7.90 (d, *J* = 7.7 Hz, 1 H), 8.16 (d, *J* = 8.2 Hz, 1 H), 8.32 (d, *J* = 2.2 Hz, 1 H), 9.20 (d, *J* = 1.5 Hz, 1 H). ^{13}C NMR ($CDCl_3$, 62.9 MHz): δ 127.1, 127.4, 128.0, 128.1, 128.6, 129.1, 129.2, 129.5, 133.3, 137.8, 147.2, 149.8. Mass m/z (%): 207 (M^++2, 14.2), 206 (M^++1, 82.6), 205 (M^+, 100.0), 176 (23.0), 151 (12.7), 102 (13.5), 77 (23.7), 55 (58.3).

6-Ethoxy-3-phenylquinoline (3j)

Yellow oil. IR (KBr): 694, 756, 825, 902, 1033, 1110, 1203, 1242, 1496, 2923, 2993, 3016 cm^{-1}. ^{1}H NMR ($CDCl_3$, 250 MHz): δ 1.51 (t, *J* = 7.0 Hz, 3 H), 4.19 (q, *J* = 7.0 Hz, 2 H), 7.12 (d, *J* = 1.0 Hz, 1 H), 7.33-7.55 (m, 4 H), 7.68-7.72 (m, 2 H), 8.03 (d, *J* = 9.2 Hz, 1 H), 8.18 (d, *J* = 1.2 Hz, 1 H), 9.01 (d, *J* = 2.2 Hz, 1 H). ^{13}C NMR ($CDCl_3$, 62.9 MHz): δ 14.7, 63.8, 106.0, 122.5, 127.4, 128.0, 128.3, 128.4, 128.1, 129.6, 130.5, 132.1, 147.3. Mass m/z (%): 251 (M^++2, 1.7), 250 (M^++1, 37.1), 249 (M^+, 57.7), 221 (100.0), 191 (16.6), 165 (40.6), 149 (21.7), 109 (46.3), 91 (30.3), 57 (46.3).

8-Methyl-3-phenylquinoline [2-5] (3k)

Yellow oil. IR (KBr): 694, 764, 902, 1033, 1072, 1380, 1450, 1496, 2916, 3021 cm^{-1}. ^{1}H NMR ($CDCl_3$, 250 MHz): δ 2.91 (s, 3 H), 7.42-7.63 (m, 5 H), 7.71-7.79 (m, 3 H), 8.38 (d, *J* = 2.5 Hz, 1 H), 9.28 (d, *J* = 2.2 Hz, 1 H). ^{13}C NMR ($CDCl_3$, 62.9 MHz): δ 18.3, 126.2, 127.3, 128.2, 128.3, 128.4, 129.3, 130.5, 133.6, 134.8, 136.1, 137.2, 144.9, 147.9. Mass m/z (%): 221 (M^++2, 13.7),

220 (M^++1, 82.4), 219 (M^+, 100.0), 189 (12.3), 165 (7.5), 140 (4.5), 115 (8.7), 89 (4.1), 55 (3.8).

8-Chloro-3-phenylquinoline [2-5] (3l)

Yellow oil. IR (KBr): 702, 763, 894, 984, 1110, 1357, 1450, 1496, 2916, 3031, 3042 cm^{-1}. ^{1}H NMR ($CDCl_3$, 250 MHz): δ 7.47-7.58 (m, 4 H), 7.70-7.74 (m, 2 H), 7.80-7.86 (m, 2 H), 8.33 (d, J = 2.2 Hz, 1 H), 9.32 (d, J = 2.2 Hz, 1 H). ^{13}C NMR ($CDCl_3$, 62.9 MHz): δ 127.0, 127.2, 127.5, 128.4, 128.5, 129.3, 129.5, 133.6, 134.7, 137.2, 143.5, 150.5. Mass m/z (%): 241 (M^++2, 4.1), 240 (M^++1, 8.7), 239 (M^+, 16.7), 205 (4.6), 104 (100.0), 78 (19.4), 57 (8.5).

References

1. Saunthwal, R. K.; Patel, M.; Verma, A. K. *J. Org. Chem.* **2016***, 81,* 6563-6572. doi: 10.1021/acs.joc.6b01186
2. Zhang, Y.; Wang, M.; Li, P.; Wang, L. *Org. Lett.* **2012**; *14*, 2206-2209. doi: 10.1021/ol300391t
3. Michael, J. P. *Nat. Prod. Rep.* **2001**, *18*, 543-559. doi: 10.1039/B005387M
4. Funayama, S.; Murata, K.; Noshita, T. *Heterocycles* **2001**, *54*, 1139-1148. doi: 10.3987/REV-00-SR(I)8
5. Sawada, Y.; Kayakiri, H.; Abe, Y.; Imai, K.; Mizutani, T.; Inamura, N.; Asano, M.; Aramori, I.; Hatori, C.; Katayama, A. *J. Med. Chem.* **2004**, *47*, 1617-1630. doi: 10.1021/jm030159x

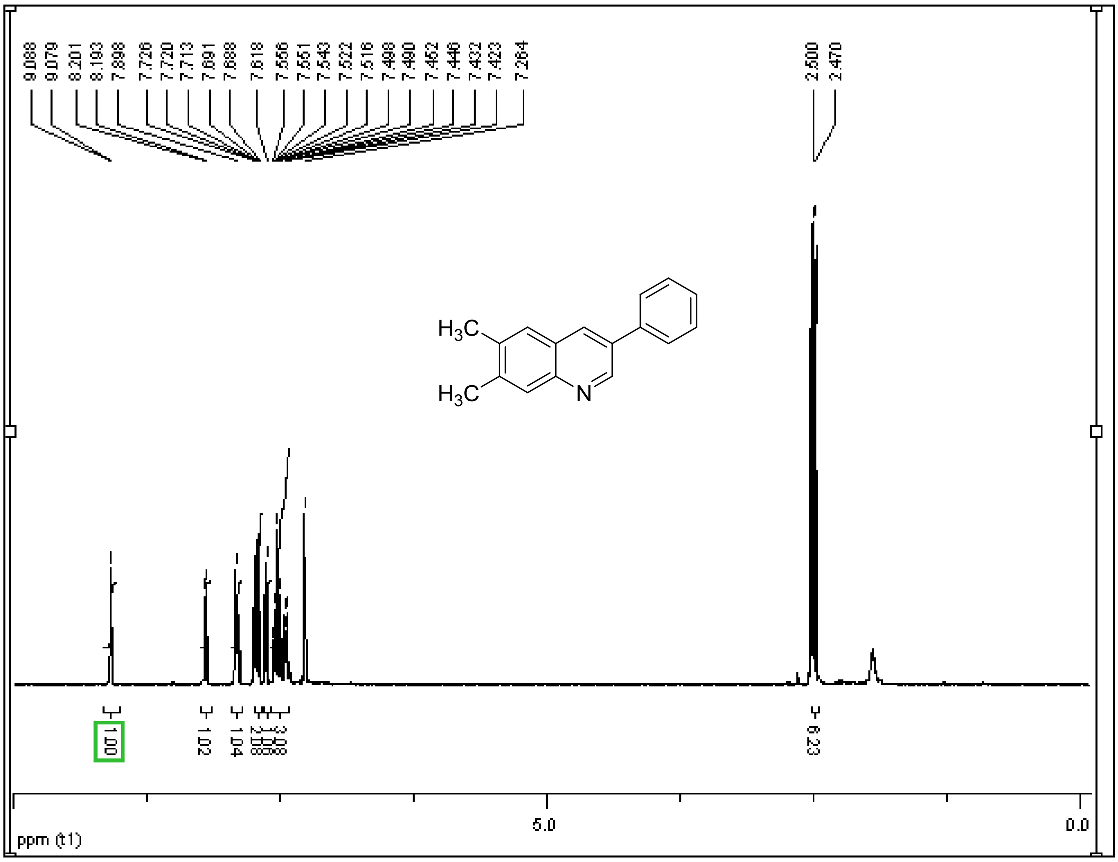

^{1}H NMR spectrum of compound **3a**

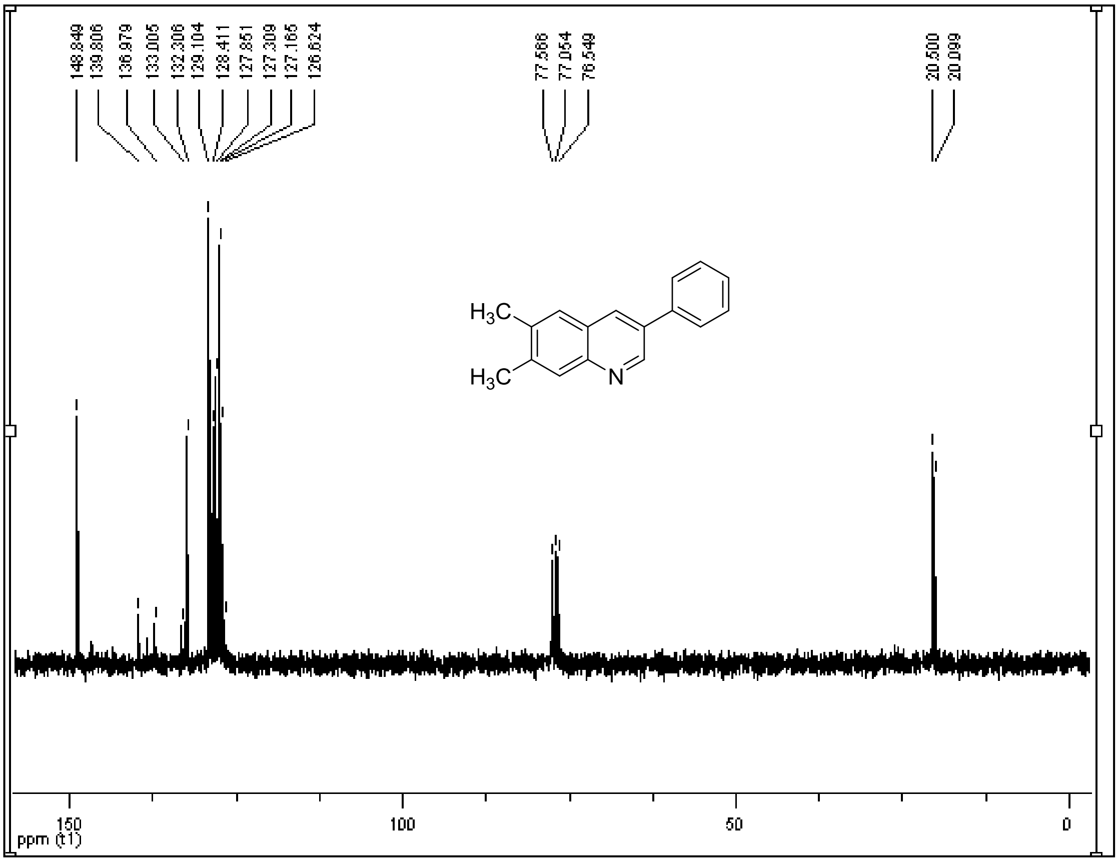

^{13}C NMR spectrum of compound **3a**

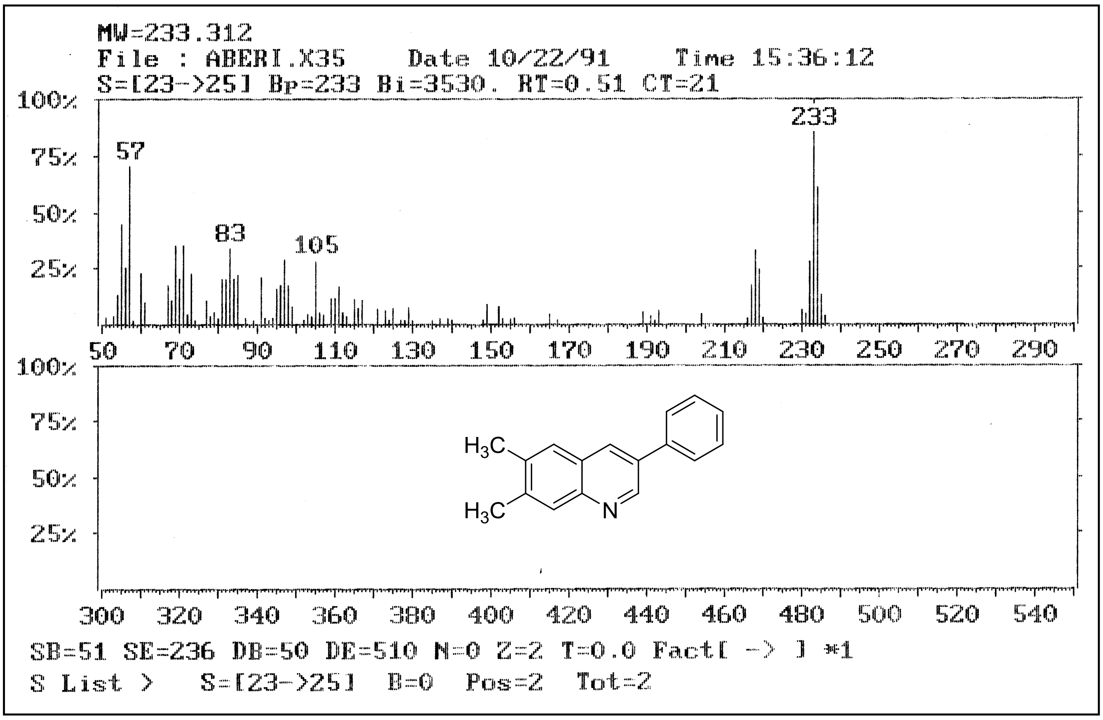

Mass spectrum of compound **3a**

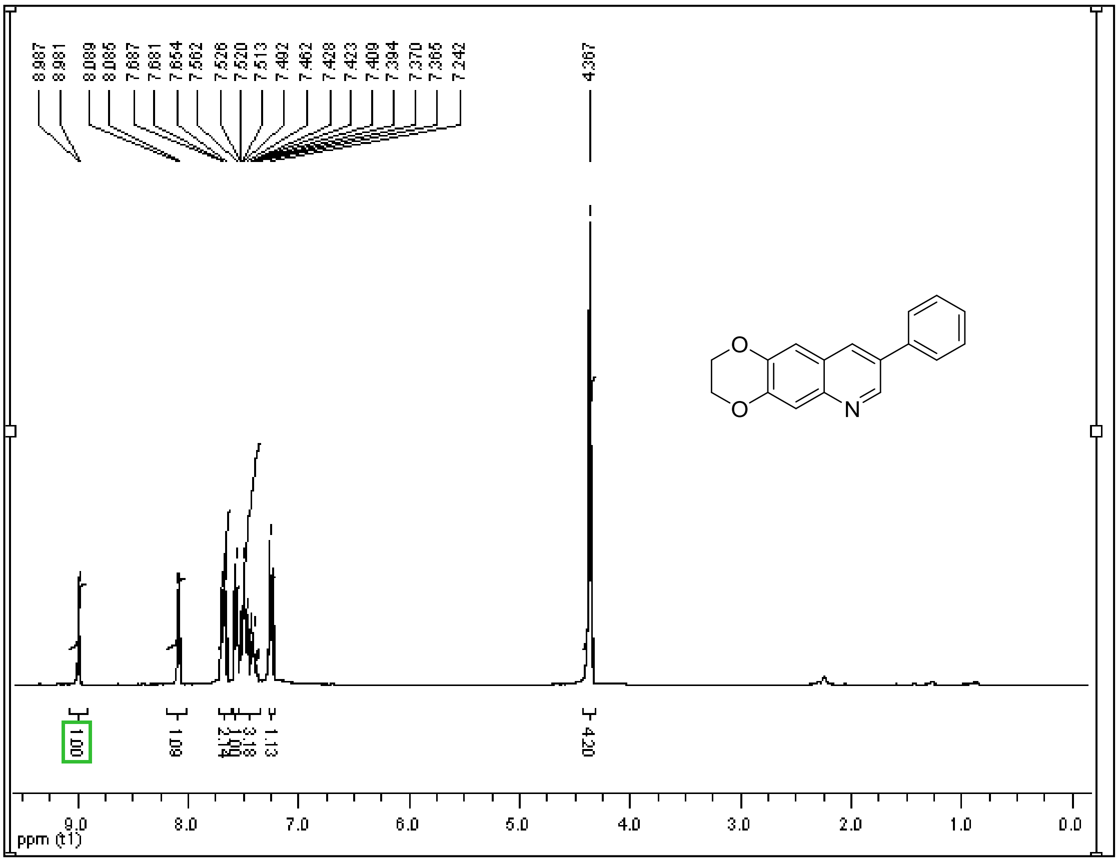

^{1}H NMR spectrum of compound **3b**

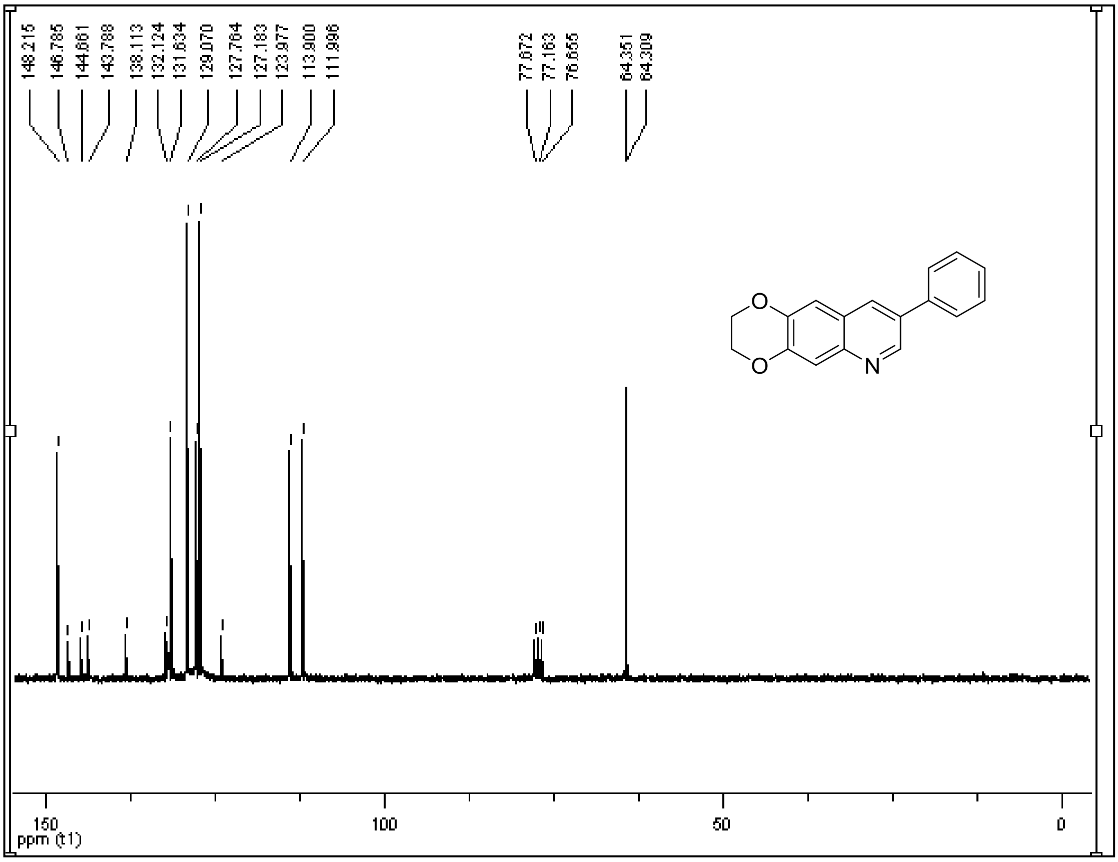

^{13}C NMR spectrum of compound **3b**

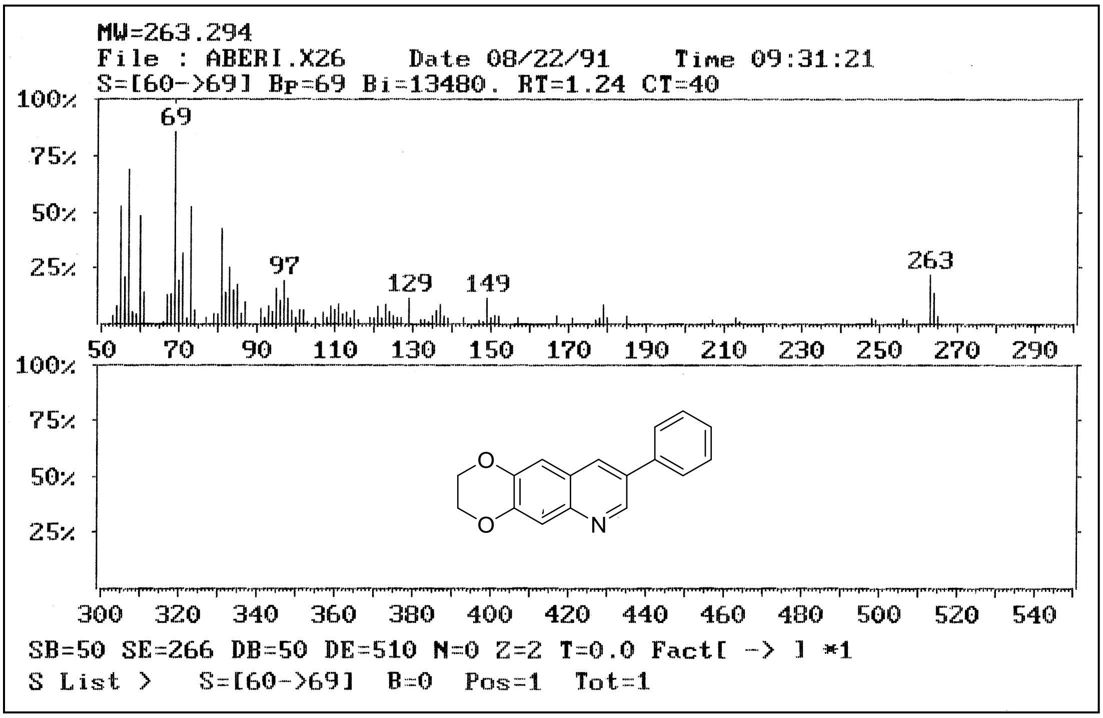

Mass spectrum of compound **3b**

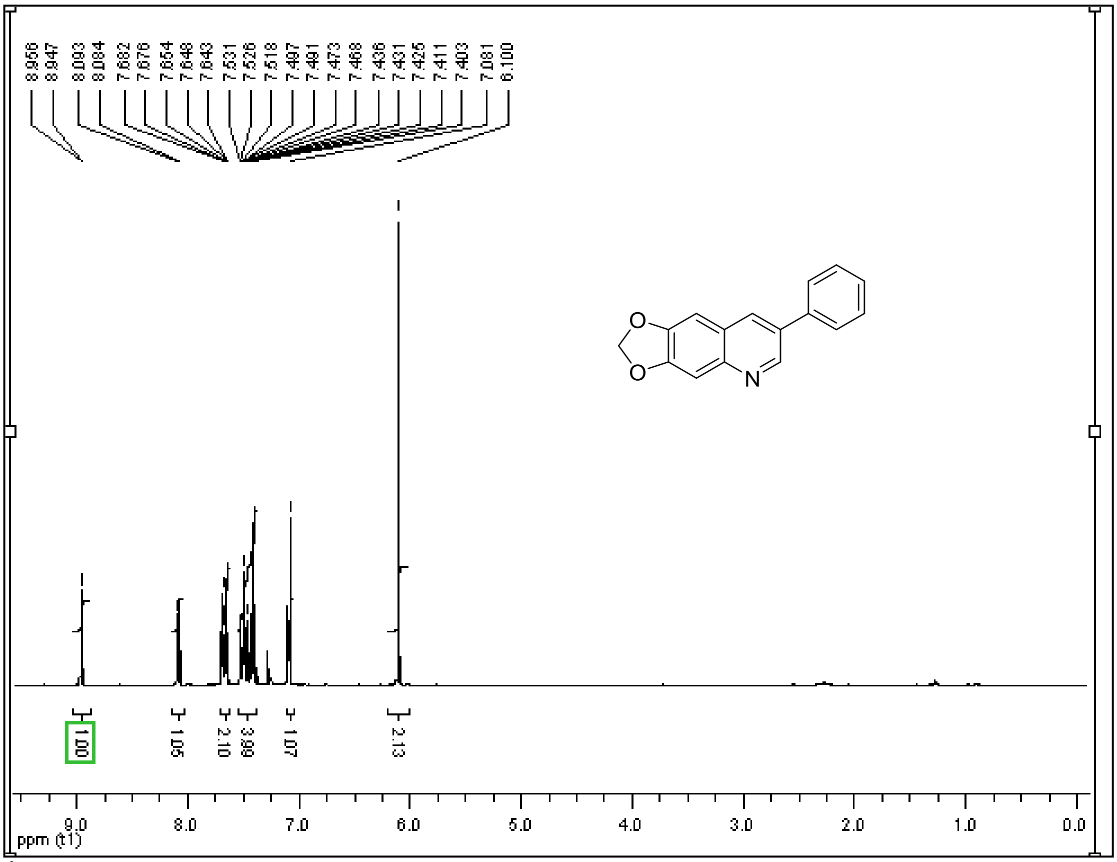

^{1}H NMR spectrum of compound **3c**

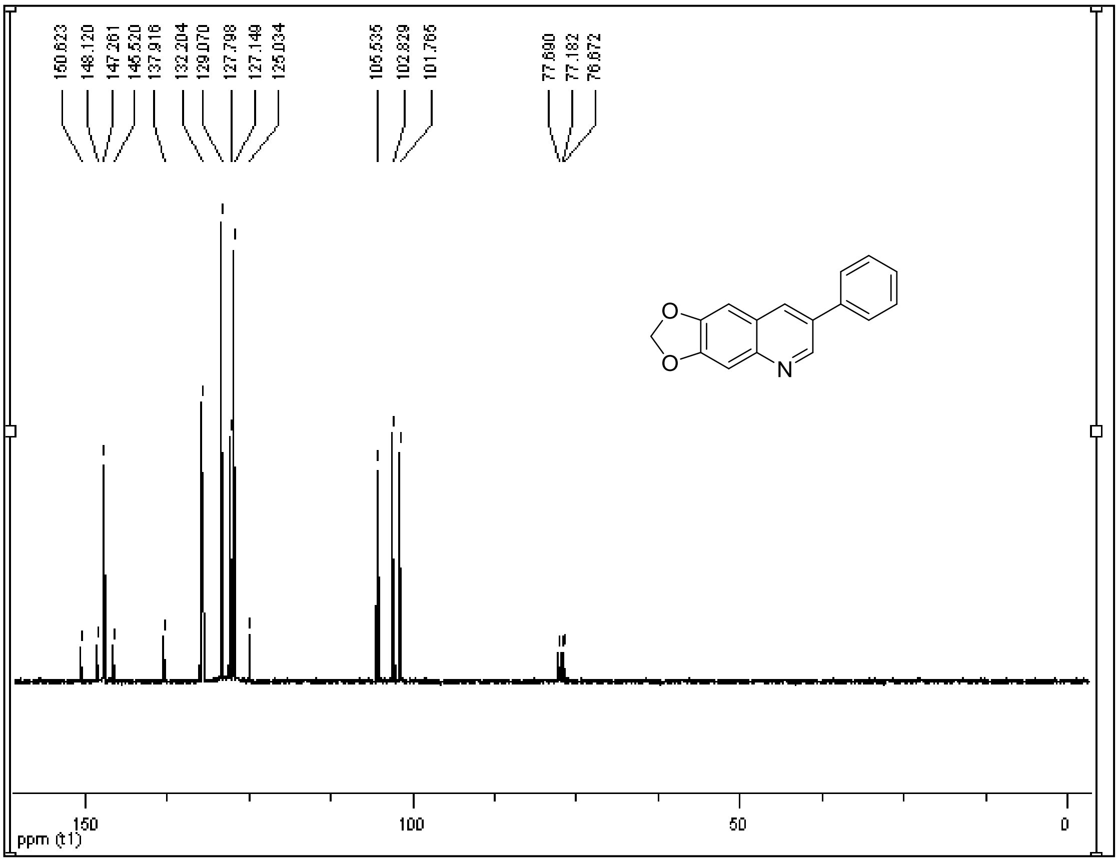

^{13}C NMR spectrum of compound **3c**

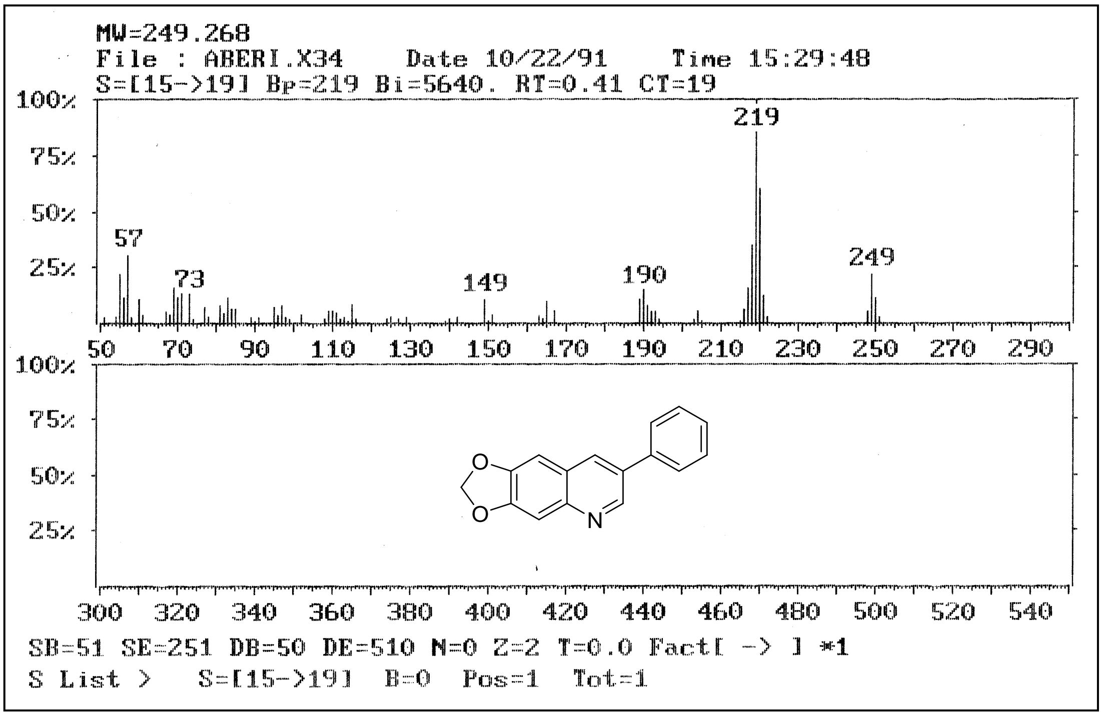

Mass spectrum of compound **3c**

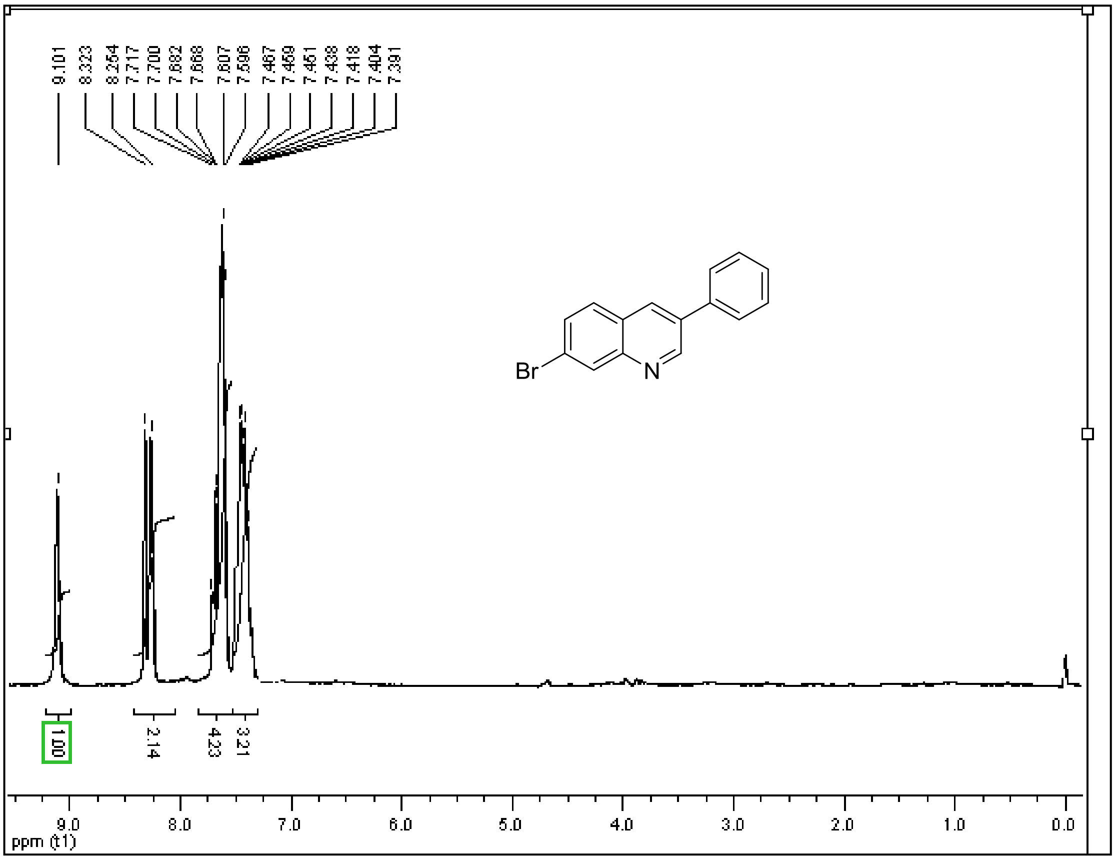

^{1}H NMR spectrum of compound **3d**

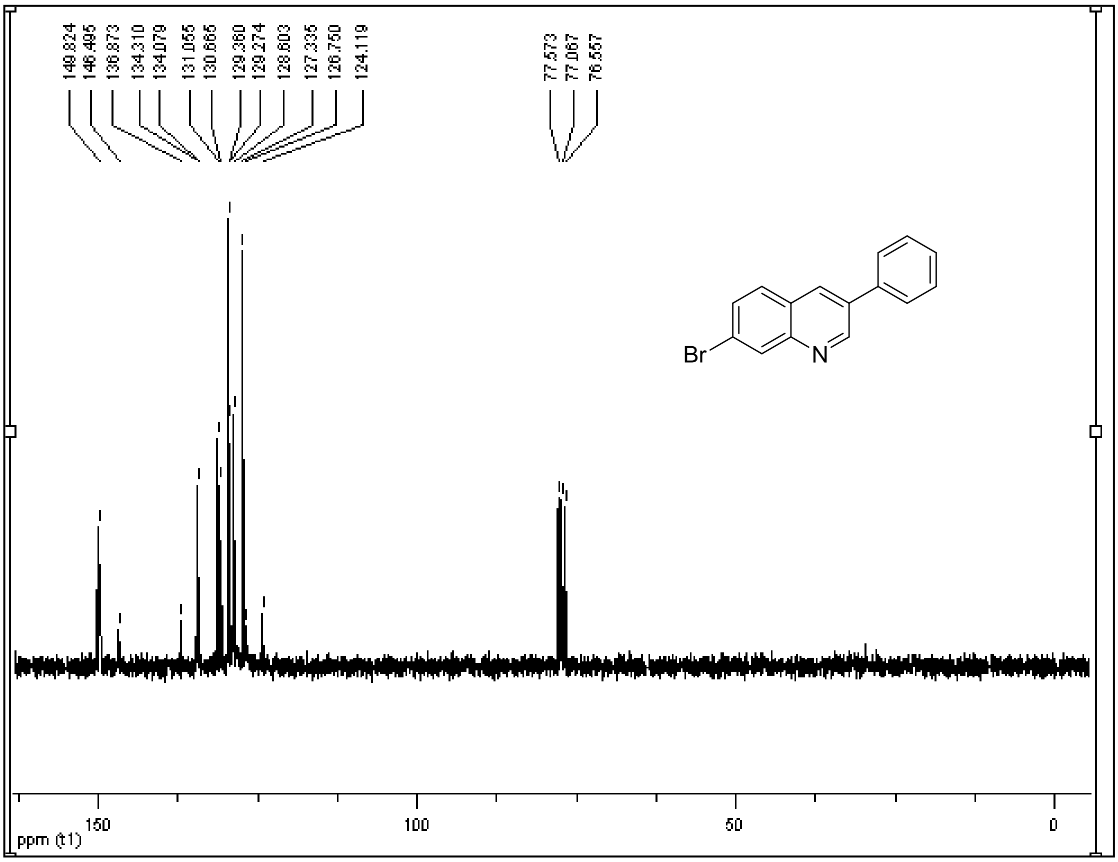

^{13}C NMR spectrum of compound **3d**

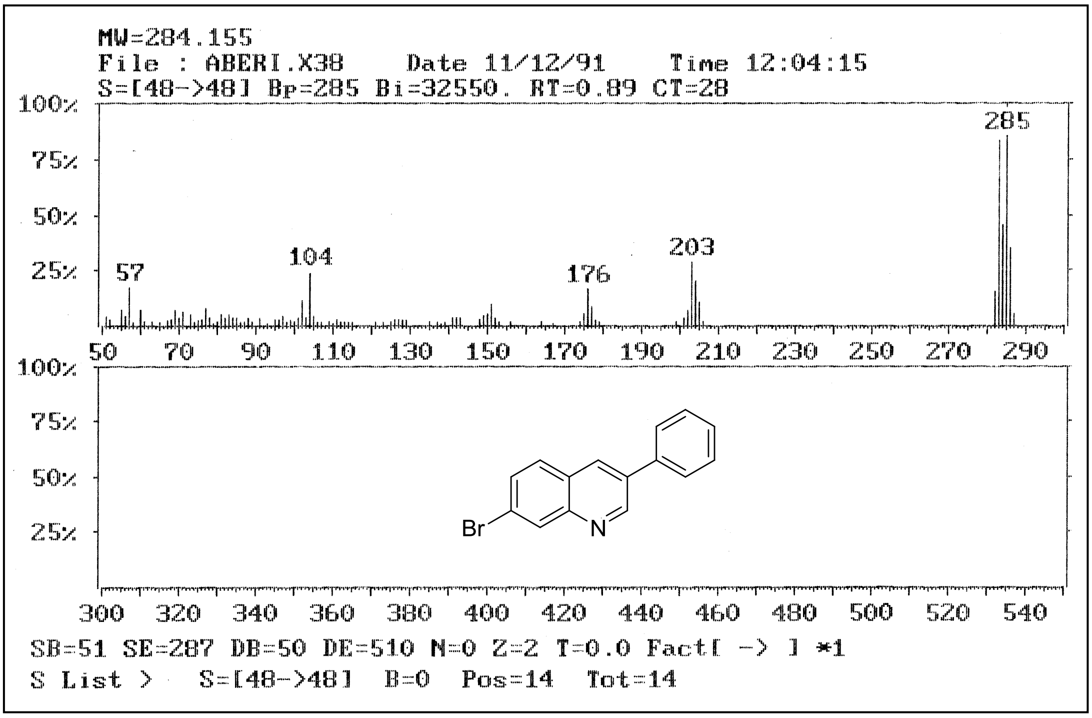

Mass spectrum of compound **3d**

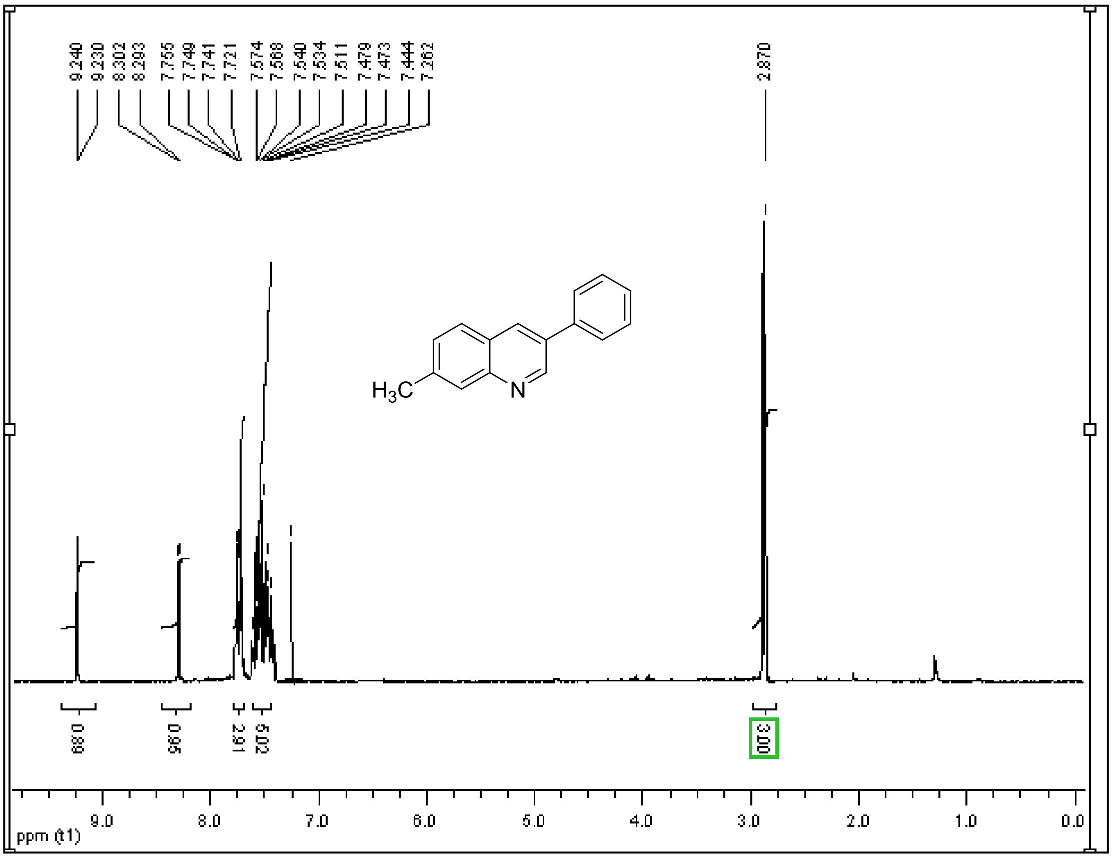

^{1}H NMR spectrum of compound **3e**

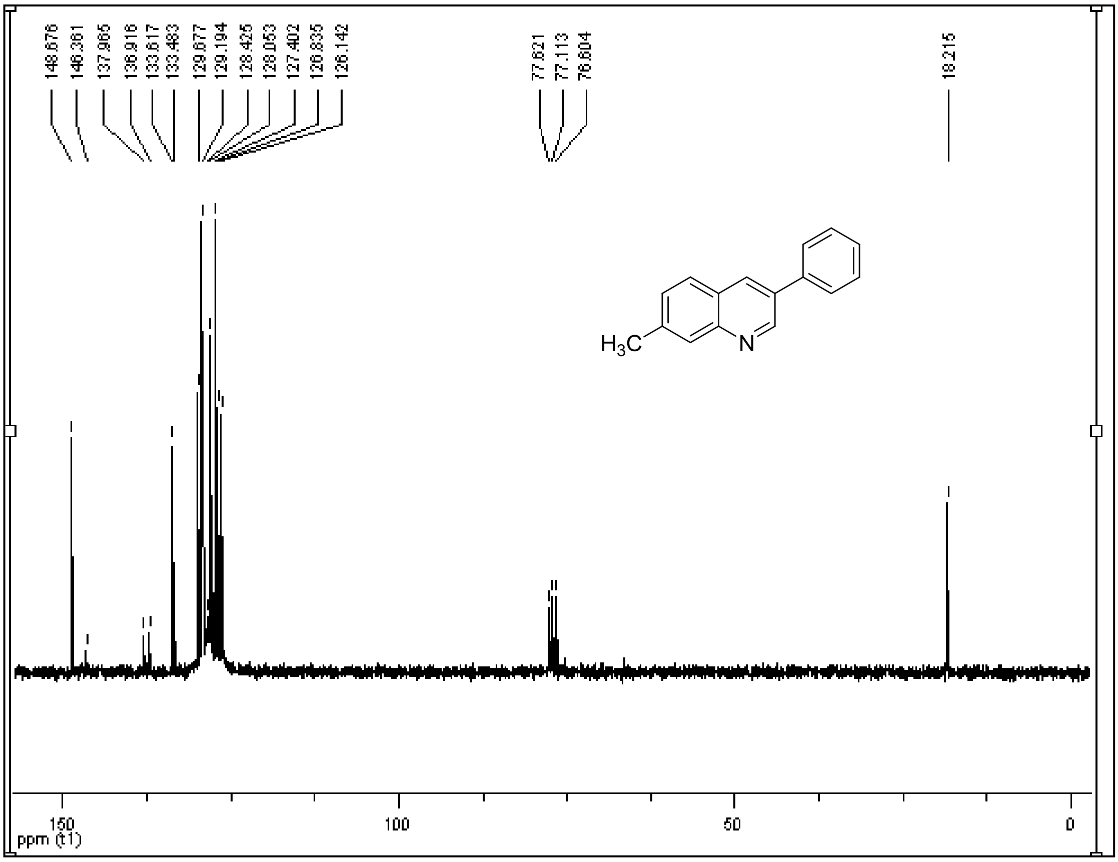

^{13}C NMR spectrum of compound **3e**

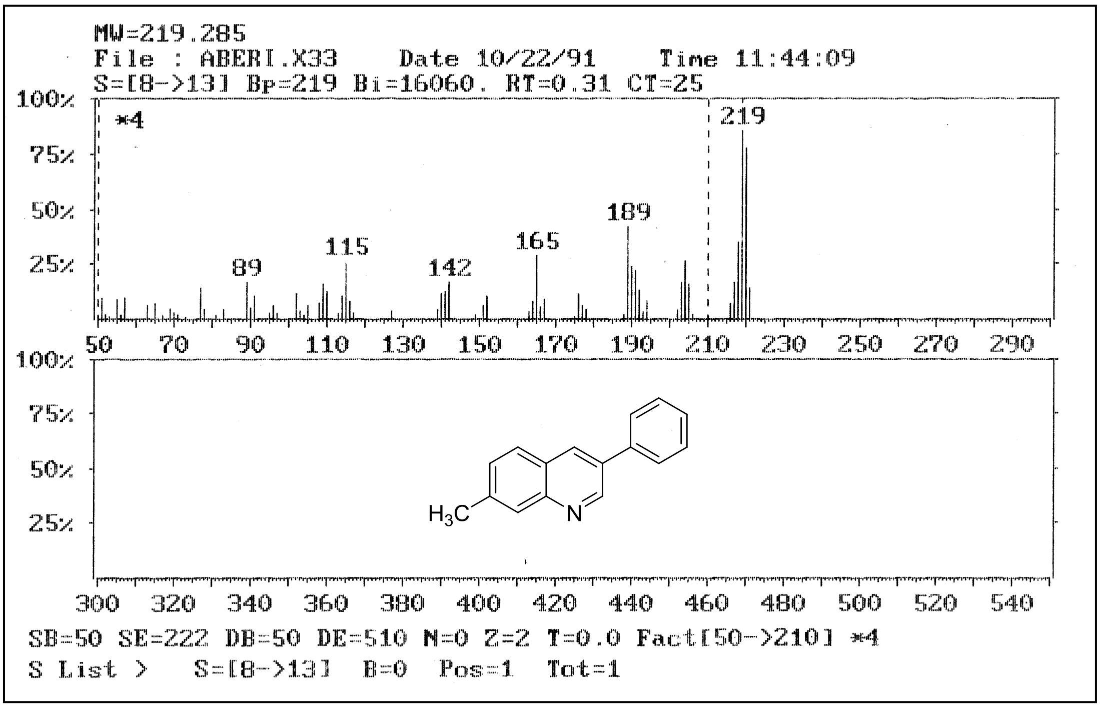

Mass spectrum of compound **3e**

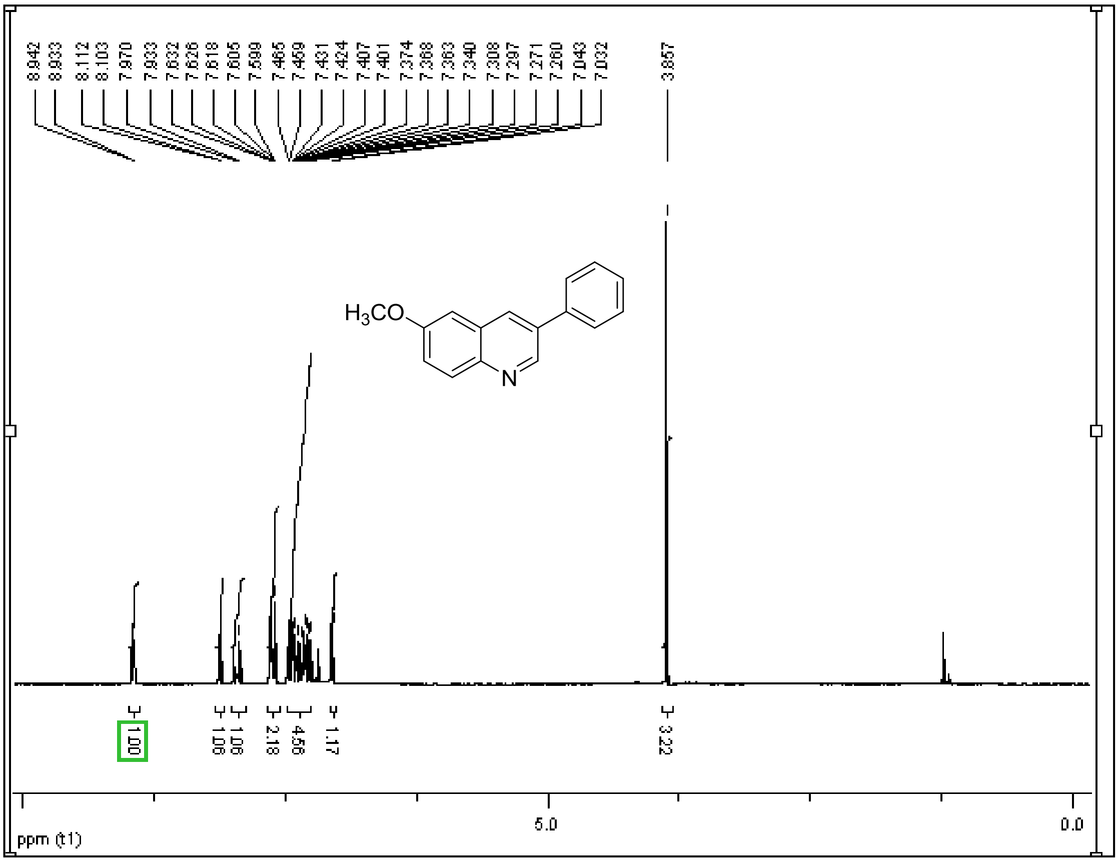

^{1}H NMR spectrum of compound **3f**

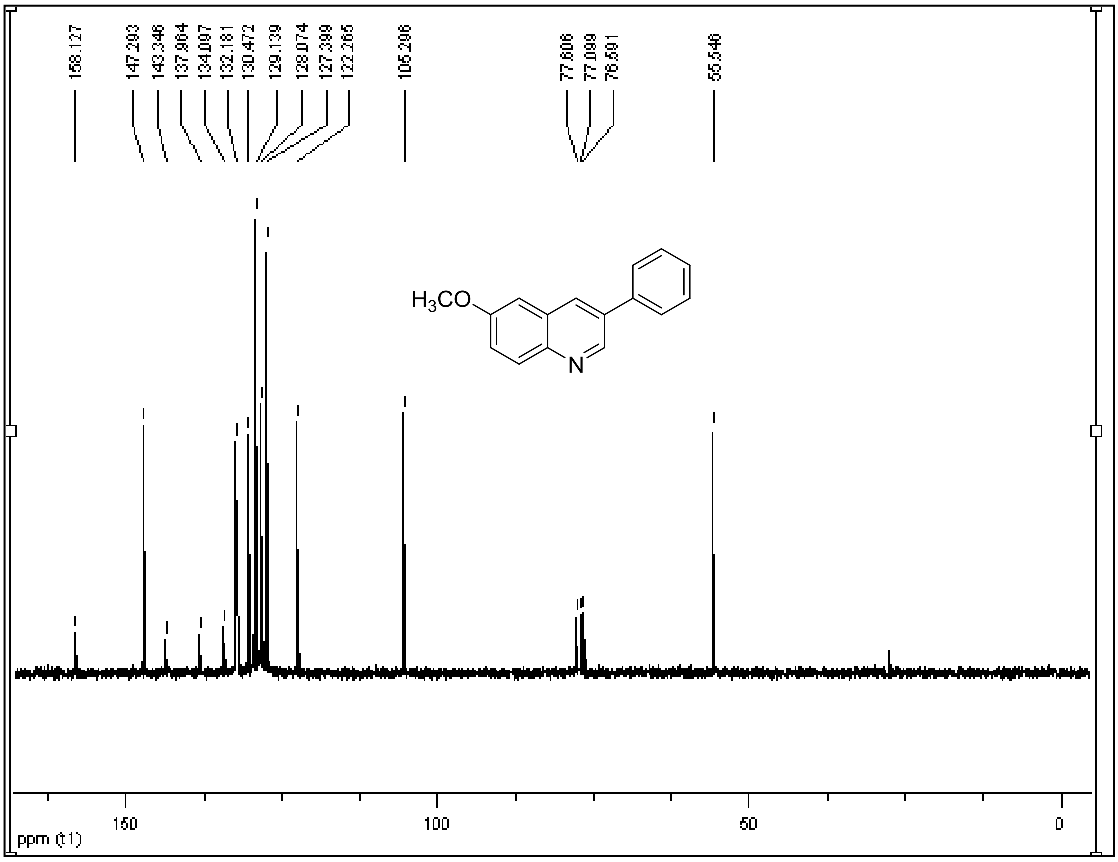

^{13}C NMR spectrum of compound **3f**

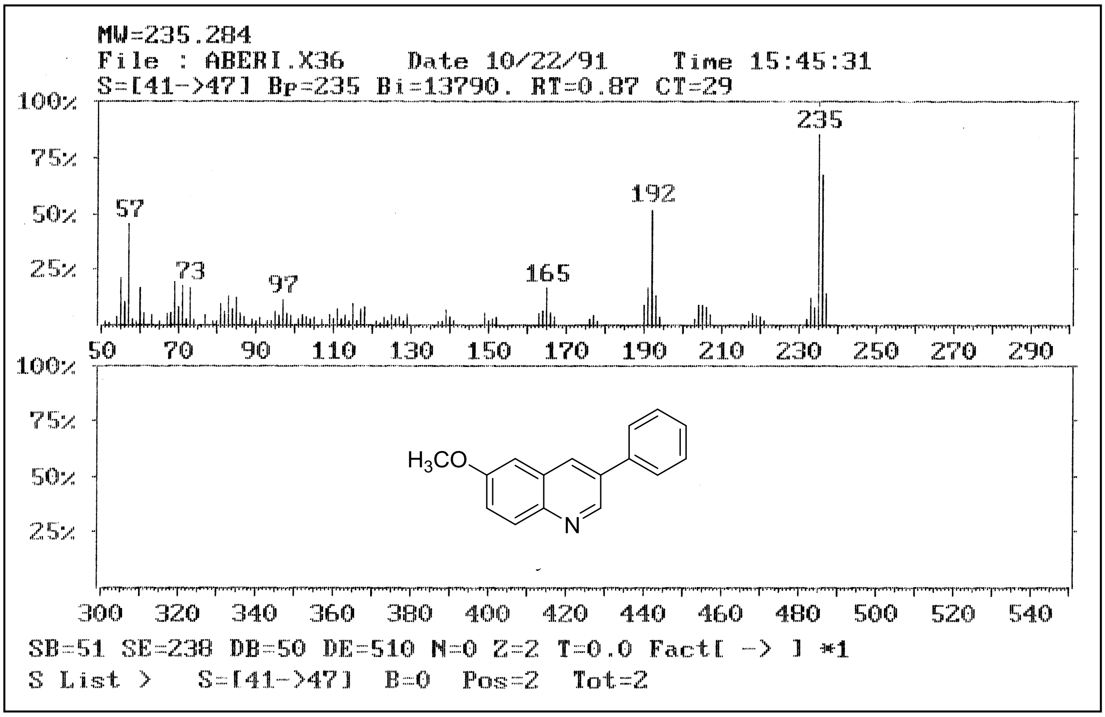

Mass spectrum of compound **3f**

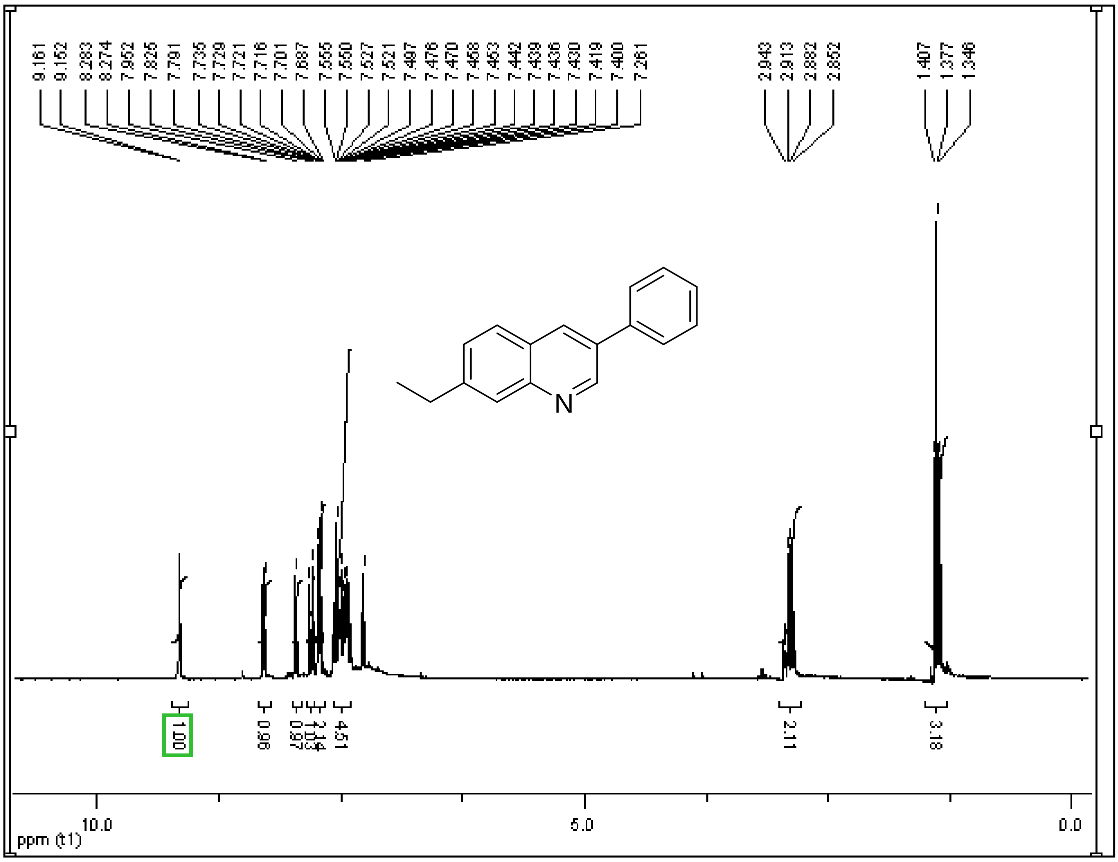

^{1}H NMR spectrum of compound **3g**

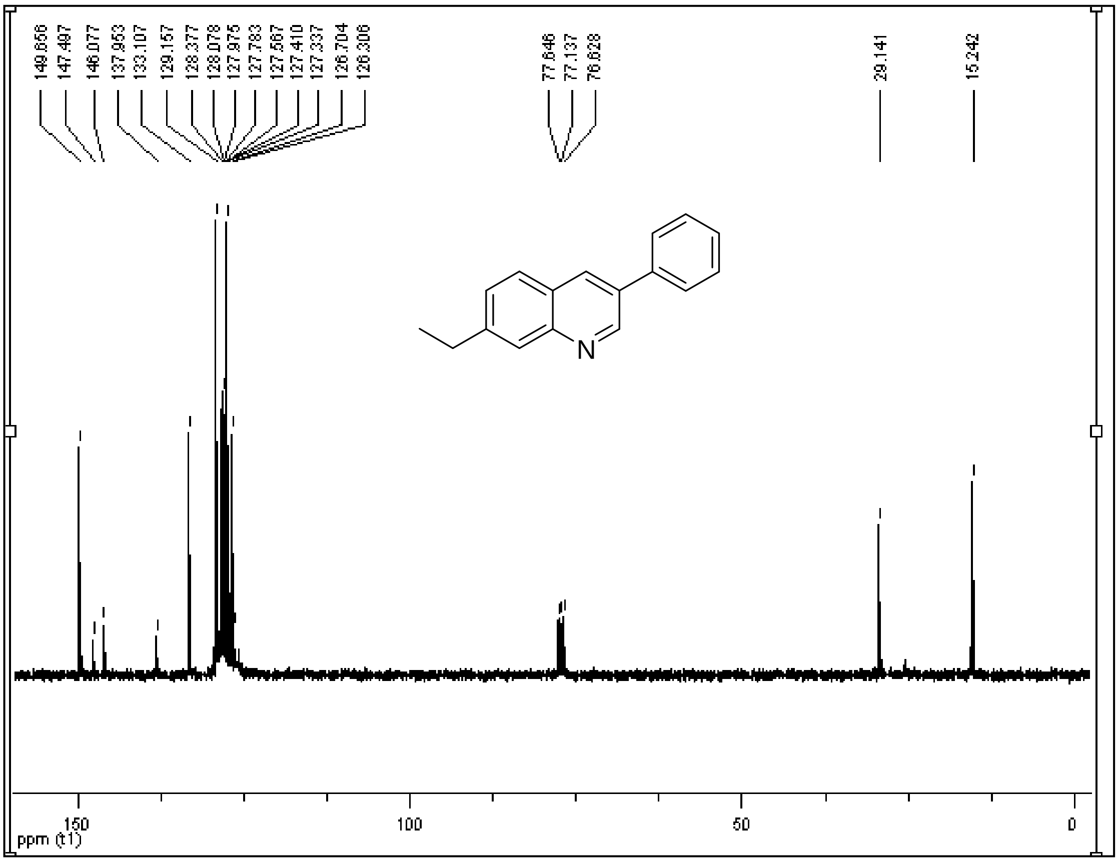

^{13}C NMR spectrum of compound **3g**

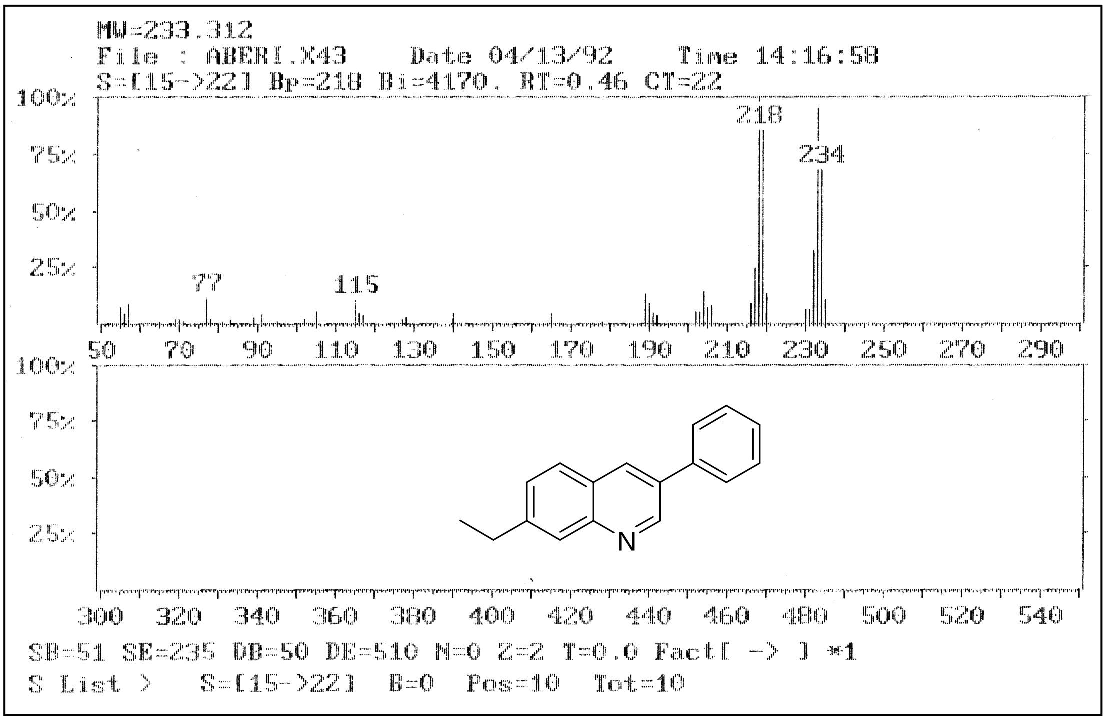

Mass spectrum of compound **3g**

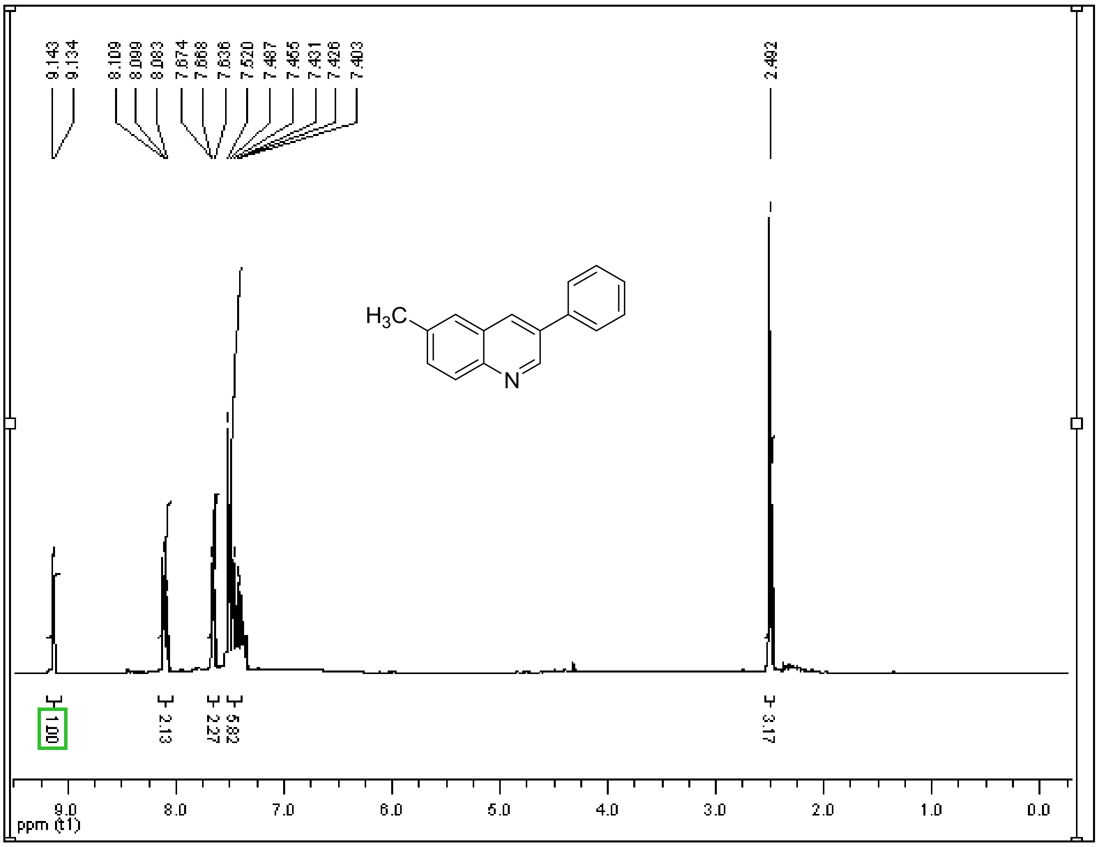

^{1}H NMR spectrum of compound **3h**

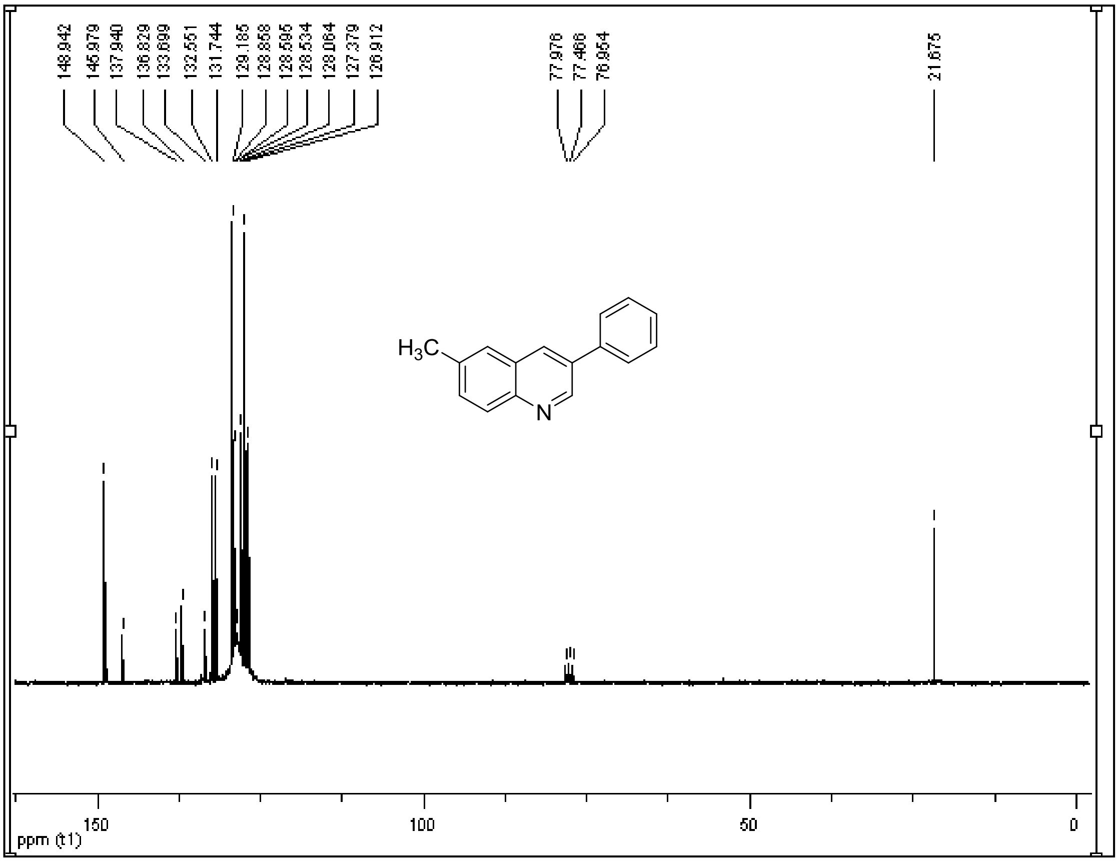

^{13}C NMR spectrum of compound **3h**

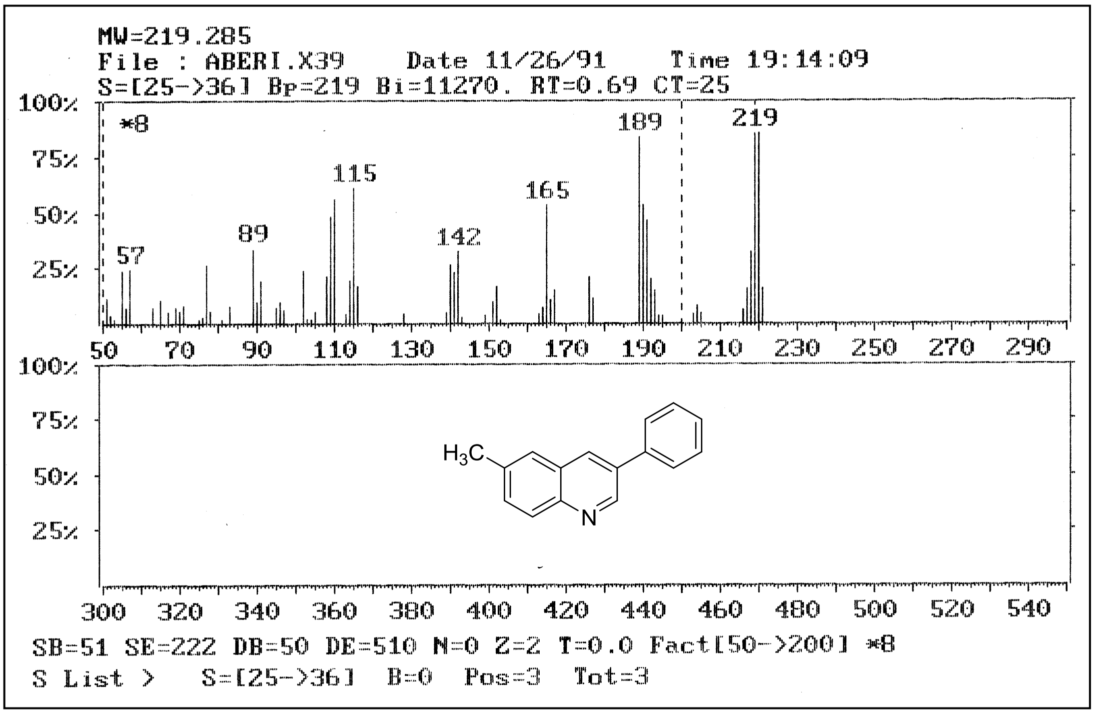

Mass spectrum of compound **3h**

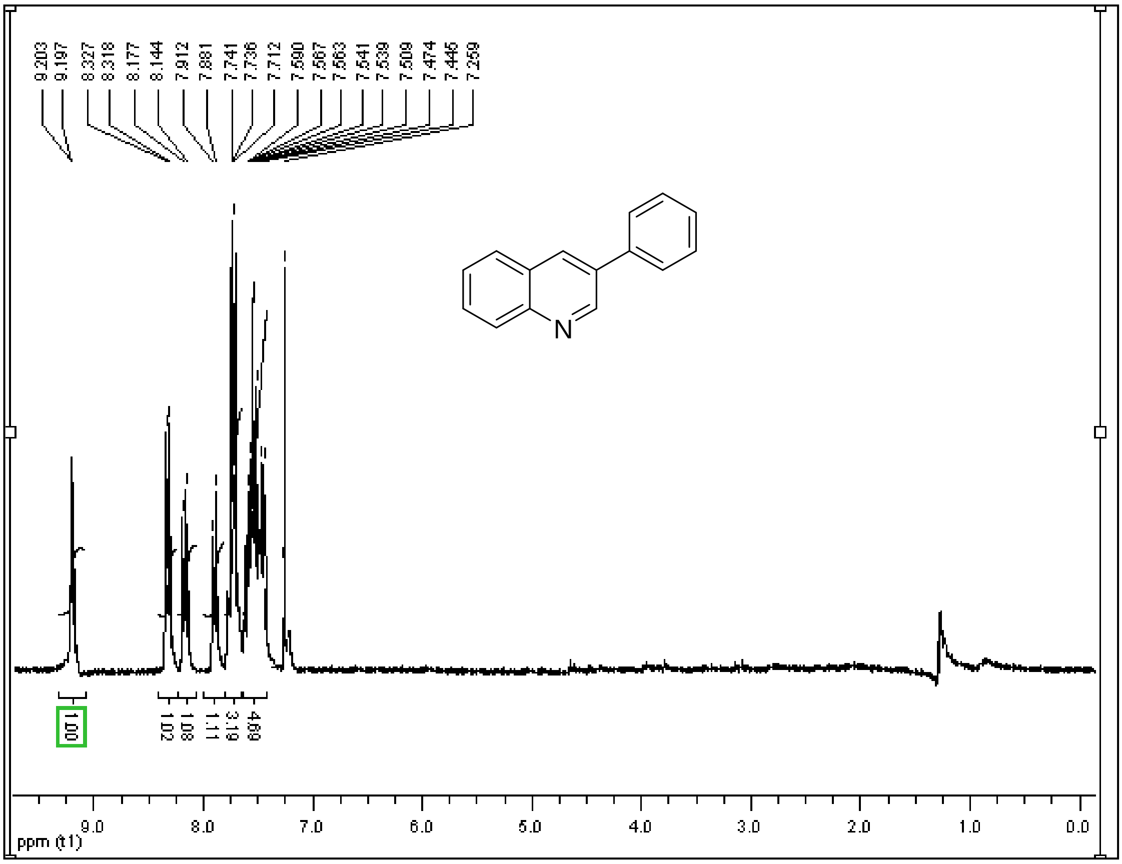

^{1}H NMR spectrum of compound **3i**

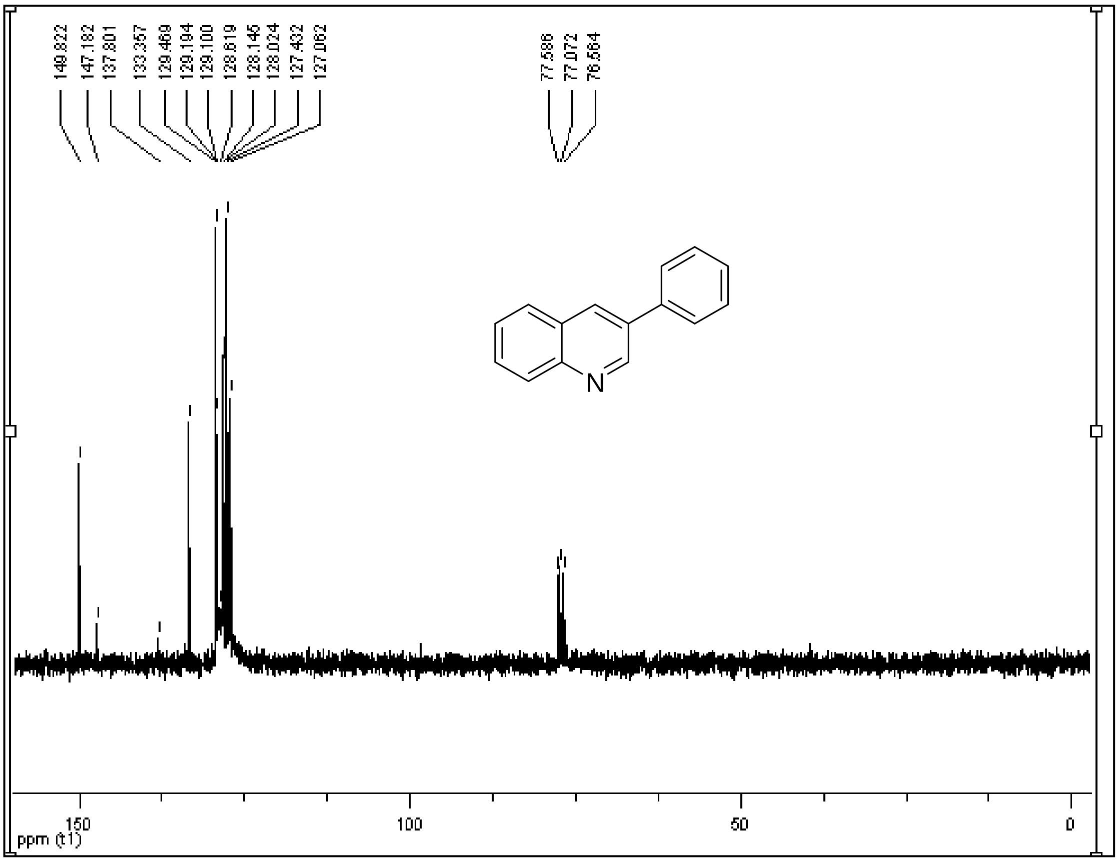

^{13}C NMR spectrum of compound **3i**

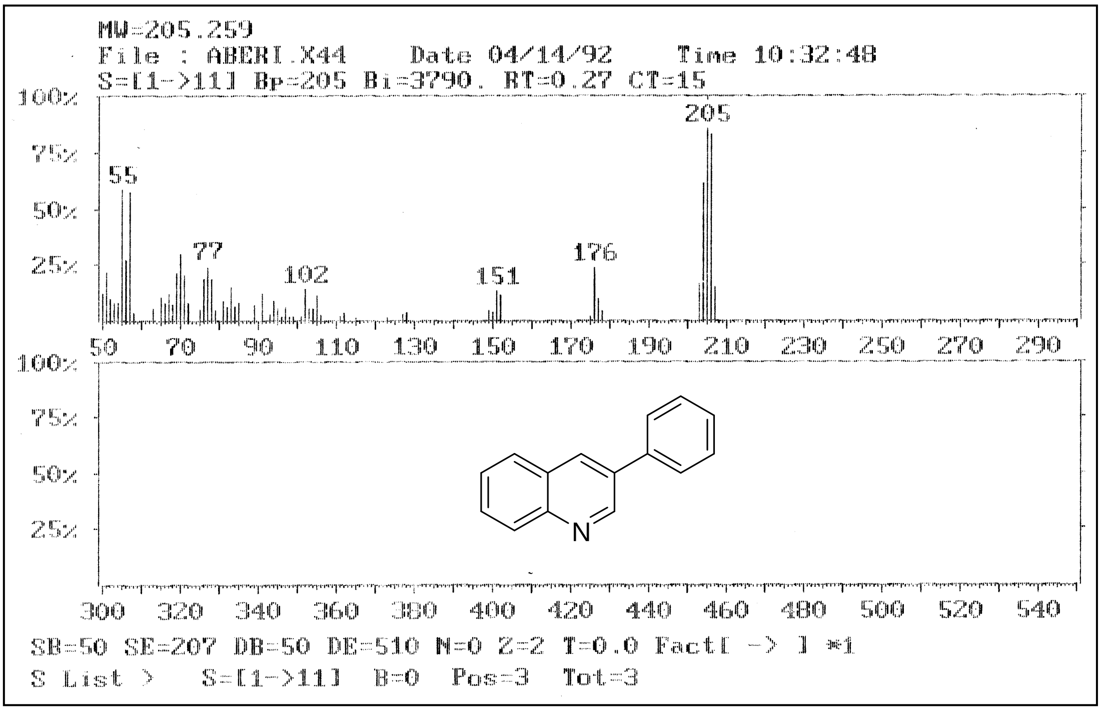

Mass spectrum of compound **3i**

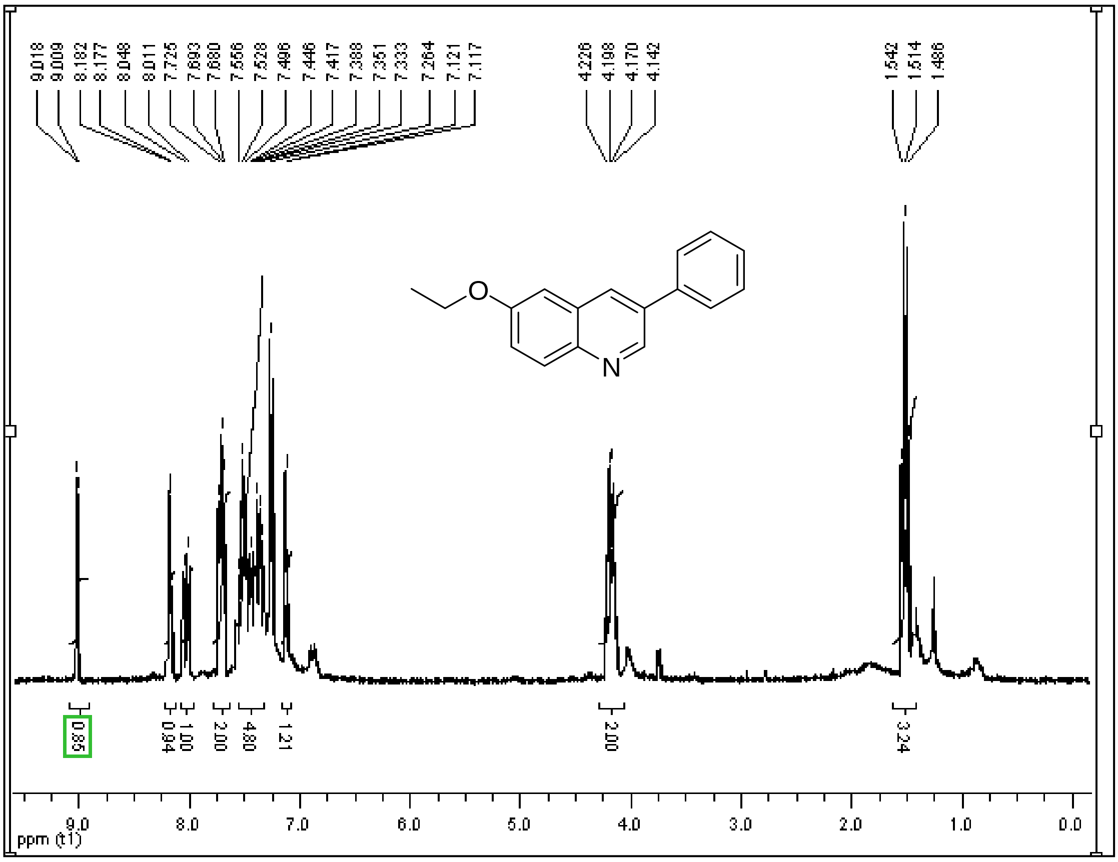

^{1}H NMR spectrum of compound **3j**

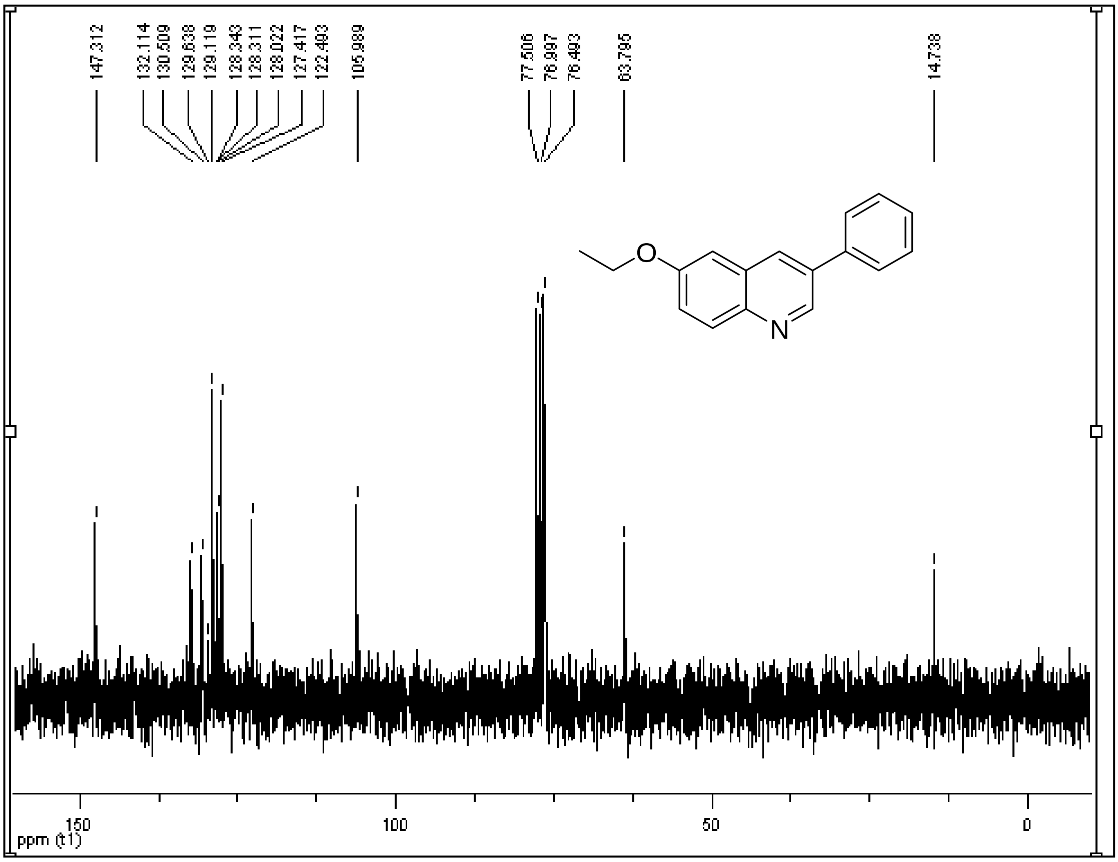

^{13}C NMR spectrum of compound **3j**

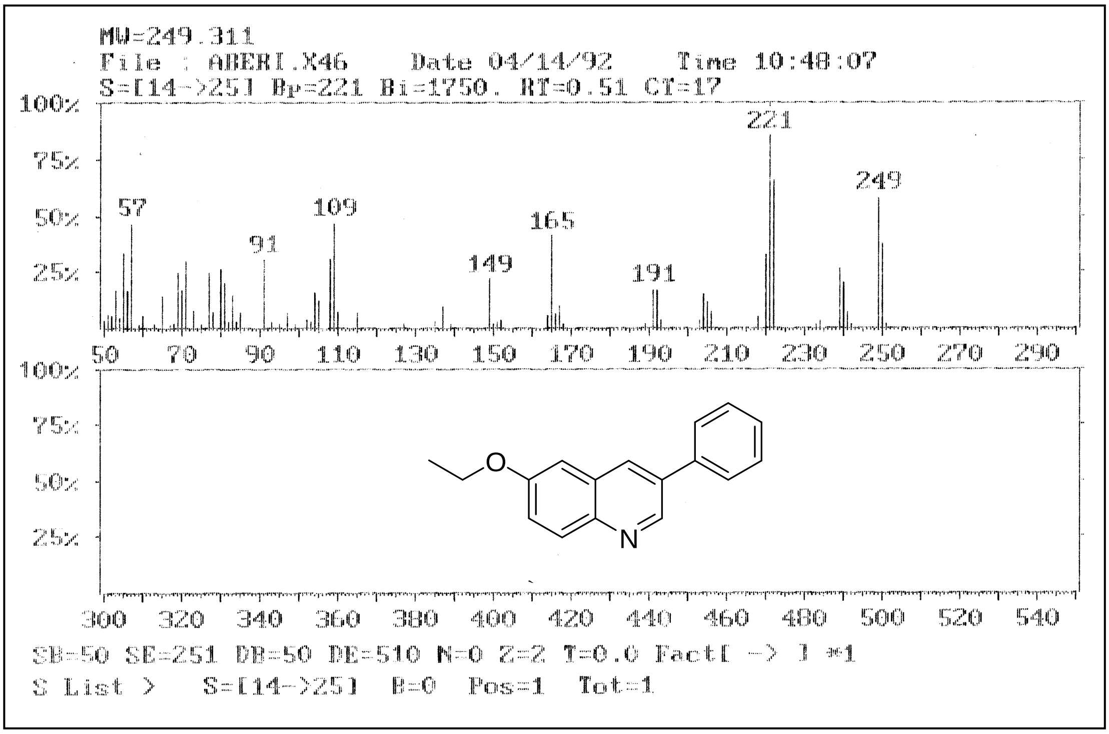

Mass spectrum of compound **3j**

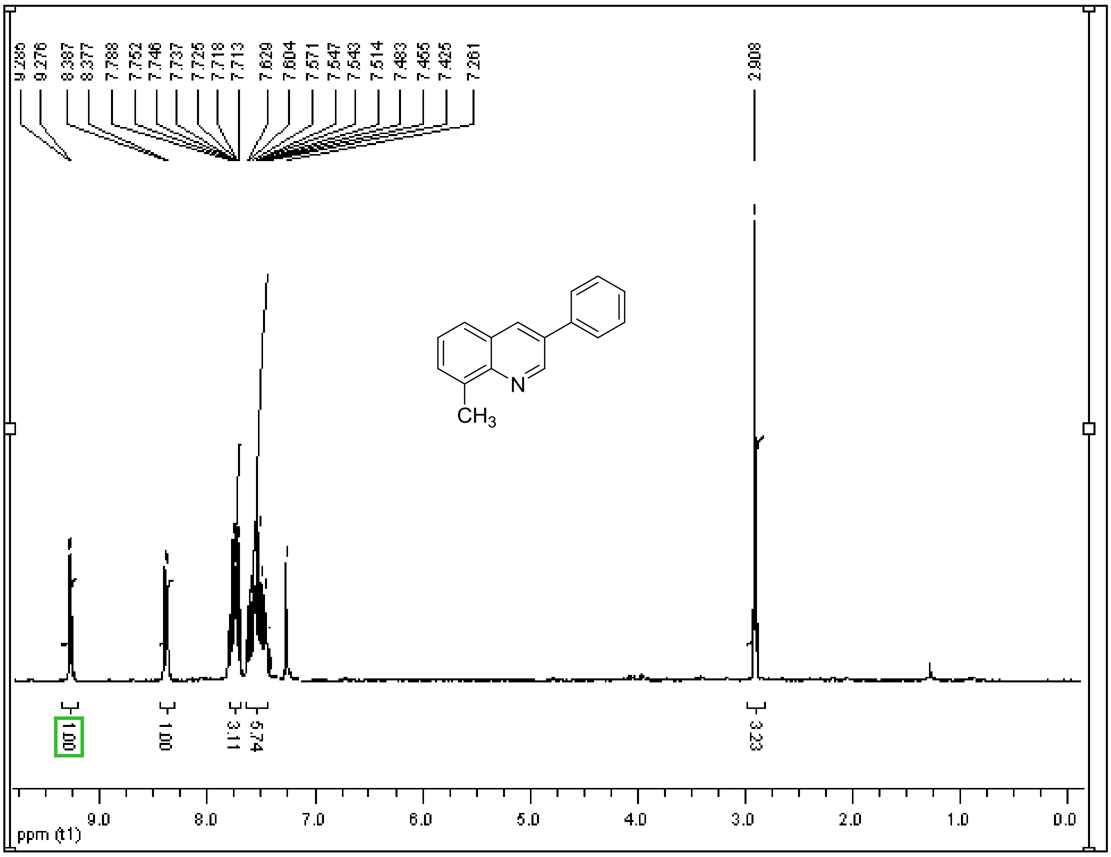

^{1}H NMR spectrum of compound **3k**

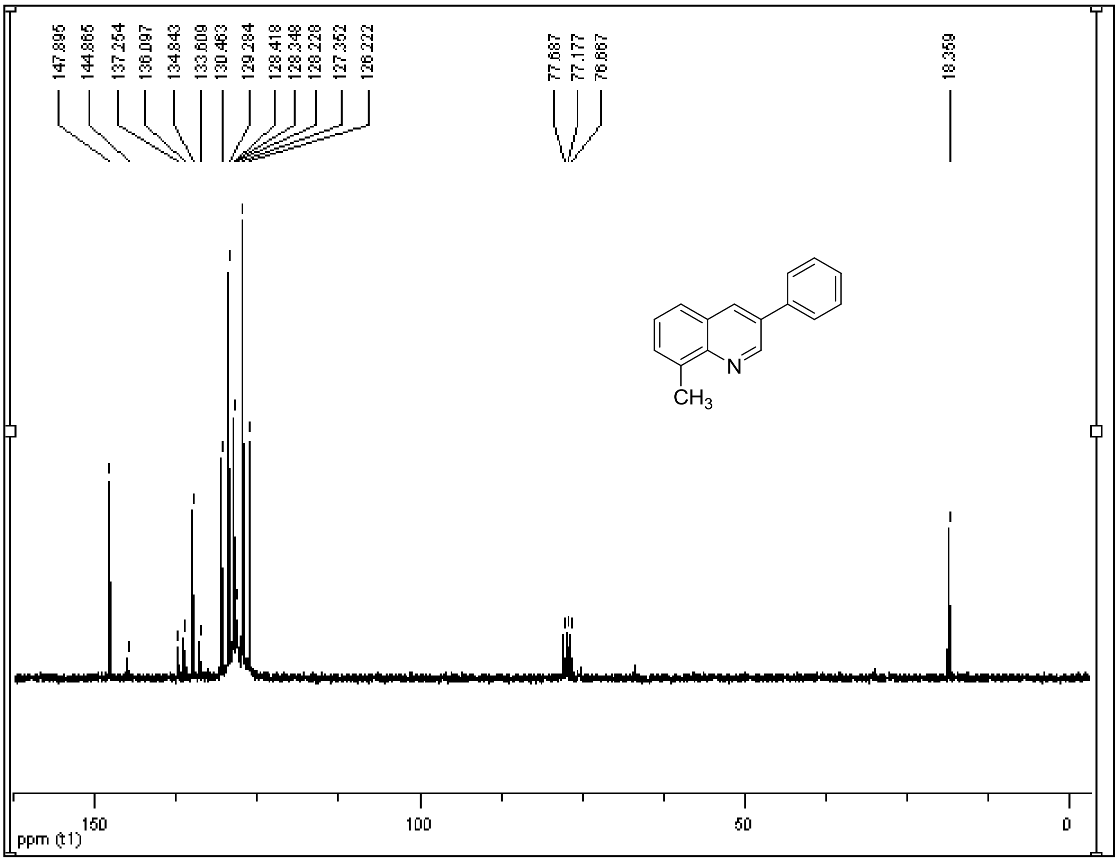

^{13}C NMR spectrum of compound **3k**

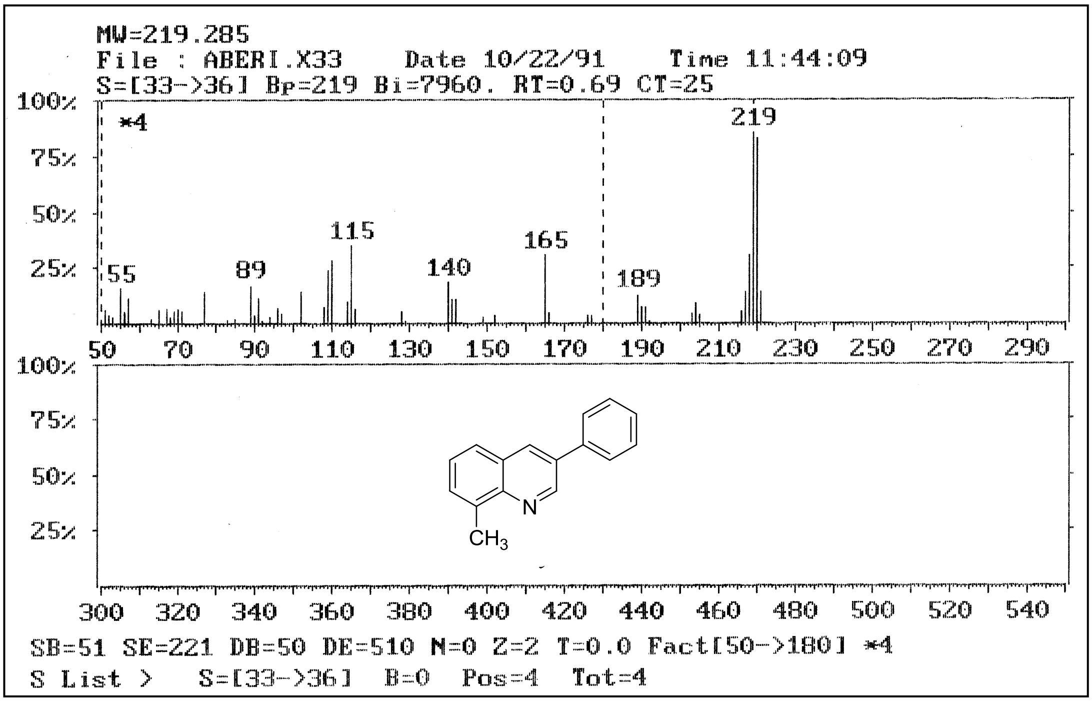

Mass spectrum of compound **3k**

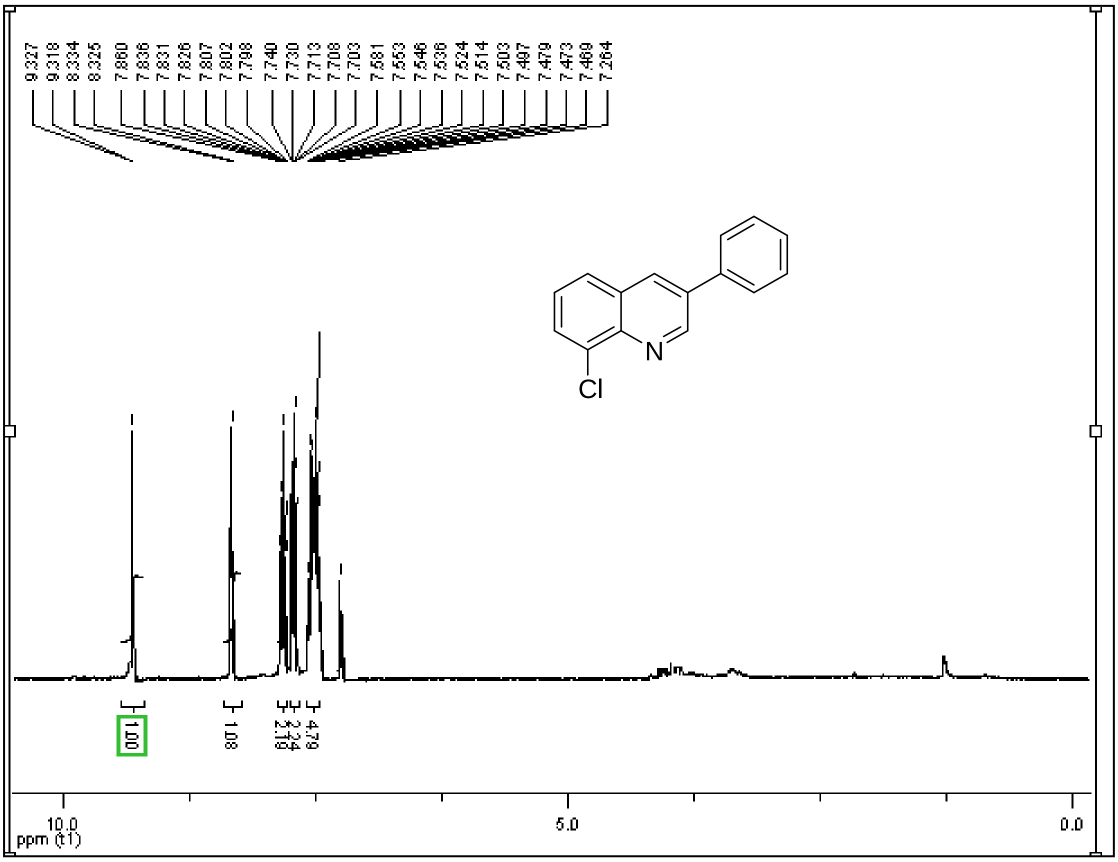

^{1}H NMR spectrum of compound **3l**

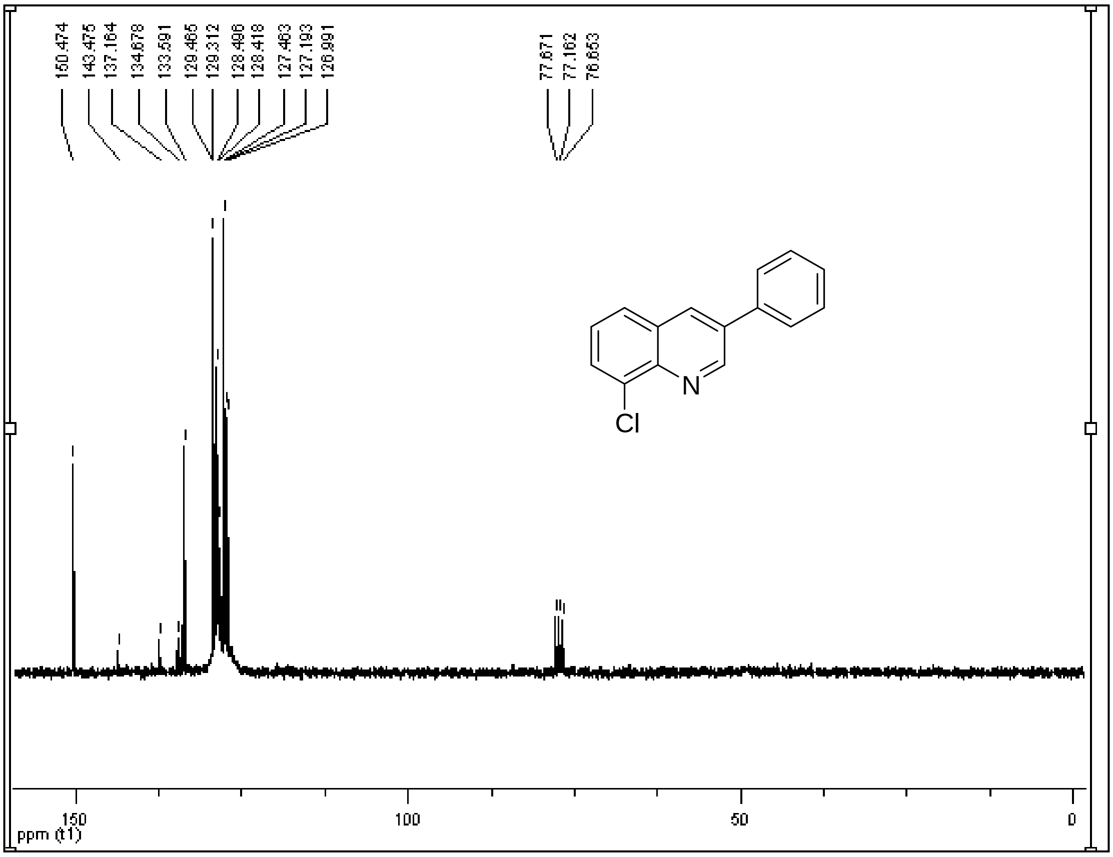

^{13}C NMR spectrum of compound **3l**

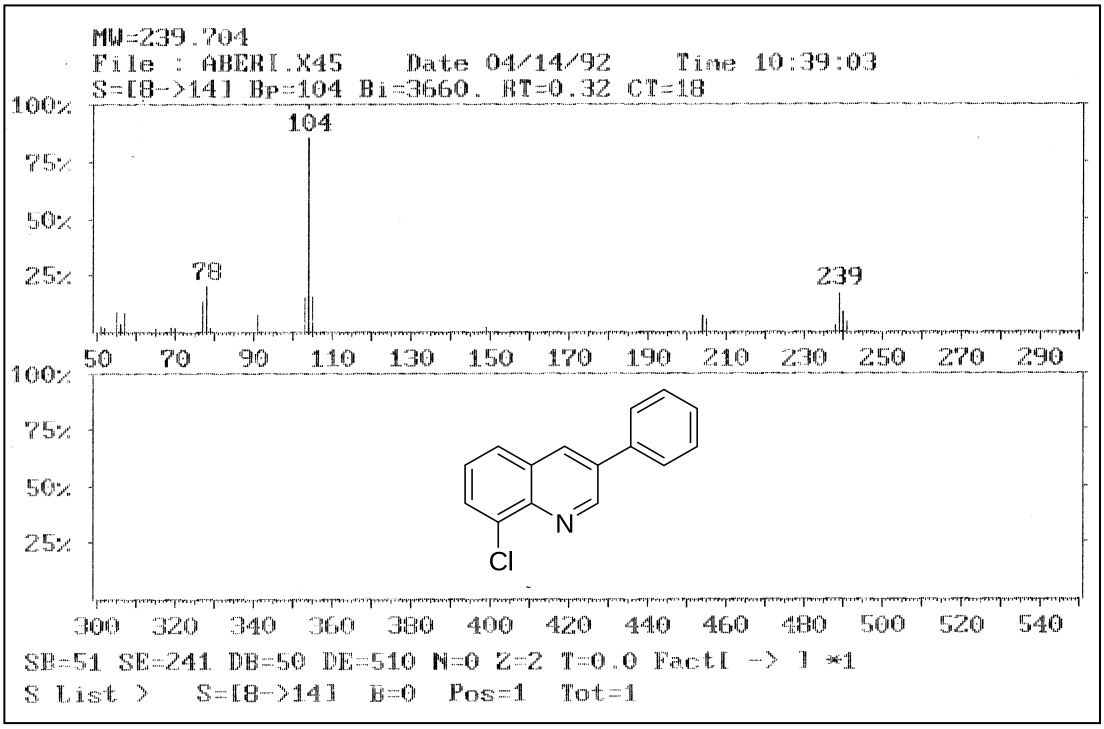

Mass spectrum of compound **3l**
